# Bcor loss promotes Richter transformation of chronic lymphocytic leukemia associated with Notch1 activation in mice

**DOI:** 10.1038/s41375-025-02557-y

**Published:** 2025-03-20

**Authors:** Chiara Rompietti, Francesco Maria Adamo, Daniele Sorcini, Filomena De Falco, Arianna Stella, Giovanni Martino, Barbara Bigerna, Erica Dorillo, Estevão Carlos Silva Barcelos, Angela Esposito, Clelia Geraci, Roberta Arcaleni, Jessica Bordini, Lydia Scarfò, Emanuela Rosati, Paolo Ghia, Brunangelo Falini, Paolo Sportoletti

**Affiliations:** 1 Department of Medicine and Surgery, Institute of Hematology and Center for Hemato- Oncology Research (CREO), University of Perugia and Santa Maria della Misericordia Hospital, Perugia, Italy; 2 IRCCS Ospedale San Raffaele, Università Vita Salute San Raffaele, Milan, Italy; 3 Department of Medicine and Surgery, Biosciences and Medical Embryology Section, University of Perugia, Perugia, Italy

**Keywords:** B-cell lymphoma, B-cell lymphoma

## Abstract

Richter’s transformation (RT) is an aggressive lymphoma occurring upon progression from chronic lymphocytic leukemia (CLL). Despite advances in deciphering the RT genetic architecture, the mechanisms driving this disease remain unknown. BCOR disruptive mutations were found in CLL and frequently associated with *NOTCH1* aberrations, a common feature in CLL and RT. We engineered mice to knock-out Bcor in B and CLL cells of Eμ-*TCL1* mice. Bcor loss resulted in alterations of the B cell compartment and favored CLL transformation into an aggressive lymphoma with reduced survival in Eμ-*TCL1* mice. RNA-sequencing demonstrated a molecular signature reminiscent of human RT and implied the involvement of the T cell tumour microenvironment in the disease onset. Bcor deficiency was associated with Notch1 activation in splenic CD19 + CD5+ cells to accelerate Eμ*-TCL1* mice lymphoproliferation. Notch1 inhibition progressively reduced circulating CD19+ CD5+ and RT cells infiltrating the spleen of diseased mice with concomitant reduction of PD-1 expressing T cells and improved survival. Our data demonstrated an interplay between the tumour suppressor activity of Bcor and Notch1 in RT pathogenesis with potential for tumour targeting. This model represented a new platform to uncover promising alternatives for this incurable tumour.

## Introduction

Richter Transformation (RT) is the occurrence of an aggressive lymphoma with poor prognosis in CLL patients. The advent of targeted therapies has improved patients’ management in CLL, whereas these advancements do not apply to RT. This also depended on the lack of preclinical models that have limited the understanding of RT pathogenesis and therapeutic developments. In these years, genome-wide sequencing deciphered the genetic architecture of CLL and RT, allowing the identification of recurrent mutations in these conditions [1–3]. Within the genetic heterogeneity of CLL, disruptive mutations of the *BCL6* co-repressor (*BCOR*) were found in up to 2% of cases [4] and have been included among the lymphoma driver genes [1]. Moreover, *BCOR* mutations frequently co-occurred with mutated *NOTCH1* [5, 6], a hallmark alteration associated with RT [7]. BCOR is a transcription factor involved in NOTCH1 signaling suppression during embryogenesis and lymphoid development [8]. It has been demonstrated that *Bcor* acts as a tumor suppressor gene by increasing the transactivating ability of NOTCH to promote leukemogenesis in mice [9]. Furthermore, *Bcor* deletion enhances nuclear accumulation of cleaved Notch1 in murine splenocytes [10], suggesting a mechanistic connection between BCOR and NOTCH1 cleavage. Here, we determined the in vivo role of *Bcor* loss in normal and leukemic B cells using Eµ*-TCL1* transgenic mice. We generated a new mouse model of RT and highlighted a pathogenic mechanism involving the interplay between Bcor and Notch1 with potential for targeted treatment.

## Methods

### Mouse strains and transplantation procedure

Experiments were performed according to murine ethical approval (authorization n°971/2020-PR – n°253/2024-PR). For all the experiments, we used homozygous *Bcor*^*flox/flox*^ female and hemizygous *Bcor*^*flox/Y*^ male mice on a C57BL/6 N genetic background [11]. *Bcor*^*flox/flox*^ and *Bcor*^*flox/Y*^ mice were intercrossed with *CD19-Cre* mice (*Cre*) [12] to generate *Bcor*^*flox/flox*^;*Cre*^+^ and *Bcor*^*flox/Y*^*;Cre*^+^ mice in order to obtain a complete Bcor loss-of-function. Hereafter, *Bcor*^*flox/flox*^;*Cre*^+^ homozygous and *Bcor*^*flox/Y*^*;Cre*^+^ hemizygous mice will be referred as *Bcor*^*−/−*^ *and Bcor*^*flox/flox*^;*Cre*^-^ and *Bcor*^*flox/Y*^;*Cre*^−^ used as controls will be indicated as *Bcor*^*+/+*^ mice. *Bcor*^*−/−*^ mice were further intercrossed with homozygous Eµ*-TCL1* (*TCL1*) models [13] to generate *Bcor*^*−/−*^*; TCL1* mice. In adoptive transfer experiments, C57BL/6 N recipients were transplanted with 2–2.5 × 10^7^ splenic cells from one original leukemic mouse donor of *TCL1* and *Bcor*^*−/−*^*; TCL1* strain, by intravenous injection (I.V.). Mice at the 2° round of transplantation were used in this study. Experiments were performed in at least three replicates using at least three original mouse donors for each strain.

### Immunophenotyping

Samples isolated from peripheral blood (PB), bone marrow (BM), spleen and liver were used for a complete blood count and immunophenotyping. The latter was conducted by flow cytometry (FC) using a 2-colors panel for the detection of CLL markers, or 3- up to 5-colors panels (supplementary Figs. 1, 2) for the characterization of B- and T-cell subpopulations in spleen and BM.

### Immunoblot analysis

Immunoblots of murine samples were performed using the monoclonal antibodies against Notch1, Hes1 and Myc.

### PCR-based analyses

- To verify the occurrence of Cre-mediated excision event of the *Bcor* locus, we performed a standard PCR on gDNA samples of splenic flow-sorted B cells, as previously described [11].
- To verify the loss of murine *Bcor* transcript, for first, and investigate the involvement of Notch1 signaling, total mRNA was extracted from splenic flow-sorted B cells, retrotranscribed, and murine *mBcor* or *mNotch1*, *mHes1* and *mMyc* expression levels, respectively, were evaluated by RT-qPCR.
- To investigate the clonality of neoplastic cells from *Bcor*^*−/−*^*; TCL1*, before and after serial transplantion, genomic DNA was isolated from splenic flow-sorted B cells and analysed for three murine IGHV families (VHJ558, VH7183, VH52Q), by standard PCR and, if necessary, Sanger sequencing.

### Histology and Immunohistochemistry

Hematoxylin and eosin (H&E) staining was performed for morphological analyses on liver and splenic sections prepared from tissues originated by non- and transplanted mice, also after Bepridil treatment. Immunohistochemistry (IHC) was performed on the same samples using the monoclonal antibody against Notch1 adopted for the immunoblot analysis. Images were acquired and interpretated with the support of an expert pathologist.

### Transcriptomic profiling

Total RNA was extracted from splenic flow-sorted B cells of transplanted mice (*N* = 3) and a precise quantity of RNA was characterized at transcriptional level by Standard RNA sequencing (RNA-Seq) with the support of Genewiz-Azenta Life Sciences. Unique Differentially Expressed Genes (DEGs) identified were used for an enrichment analysis conducted by an expert bioinformatician.

### Drug administration

Three C57BL/6 N female recipients per treatment arm (group #1: Vehicle; group #2: Bepridil) were I.V. injected with 2–2.5 × 10^7^ splenic cells from three original leukemic mouse female donors of Bcor^−/−^; TCL1 strain (leukemic burden >50%). Transplanted mice were injected intraperitoneally with DMSO (vehicle) or bepridil (5 mg/kg), respectively, once daily, 5 days/week, over a 1-month period. Only mice at second round of transplantation received the treatment.

### Additional methods

Further details on mouse models, experimental conditions, bioinformatic analyses, reagents and instrumentation information are provided in supplementary materials

## Results

### Bcor deficiency perturbs B cell differentiation but is not sufficient to drive lymphoid malignancies in mice

We crossed homozygous *Bcor*^*flox/flox*^ female mice and hemizygous *Bcor*^*flox/Y*^ male mice with *CD19-Cre* mice (*Cre*+) to obtain a complete deletion of Bcor in B cells. Both *Bcor*^*flox/flox*^;*Cre*^+^ homozygous *(Bcor*^*−/−*^) and *Bcor*^*flox/Y*^*;Cre*^*+*^ *(Bcor*^*−/−*^) hemizygous mice were characterized by a complete Bcor loss-of-function. Analysis of splenic B cells confirmed the deletion of Bcor at DNA, mRNA and protein levels (supplementary Fig. 3A). Flow cytometric analyses of *Bcor*^*−/−*^ splenocytes revealed a significant 1.35-fold reduction of total B220+ cells compared with *Bcor*^*+/+*^ mice (35.13 × 10^6^ ± 11.56 *vs* 47.57 × 10^6^ ± 15.34 cells, respectively; supplementary Fig. 3B) along with an altered distribution of B-cell subpopulations, characterized by a 1.6-fold reduction of the follicular (Fo) population (8.76 × 10^6^ ± 5.78 *vs* 14.03 × 10^6^ ± 10.74 cells; supplementary Fig. 3C) and a 1.45-fold increase of marginal zone (MZ) B cells (1.58 × 10^6^ ± 1.05 *vs* 1.05 × 10^6^ ± 0.68 cells, respectively; supplementary Fig. 3D). BM mature recirculating B cells were 1.6-fold reduced in *Bcor*^*−/−*^ mice than controls (1.69 × 10^6^ ± 0.69 *vs* 2.69 × 10^6^ ± 1.47 cells, respectively; supplementary Fig. 3E).

During a 18-months follow up, *Bcor*^*−/−*^ mice did not present alterations in PB counts (supplementary Fig. 4A), in the number of CD19+ CD5+ cells (79.83 ± 41.9 *vs* 72.85 ± 41.31 cells/µL of *Bcor*^*+/+*^*;* supplementary Fig. 4B) or sign of lymphoid disease leading to death (supplementary Fig. 4C), suggesting that Bcor deficiency is insufficient to drive lymphoid malignancies in mice.

### Bcor loss sustains transformation of Eμ-*TCL1* leukemia toward a high-grade lymphoid malignancy mimicking human RT

**Fig. 1:**
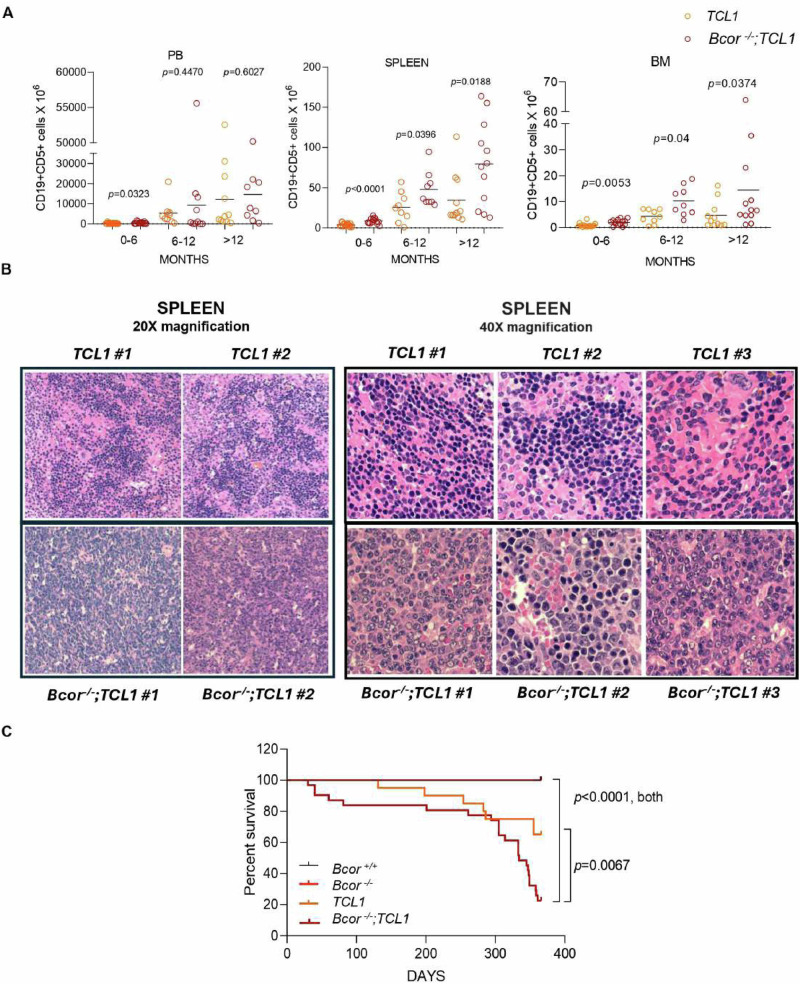
Bcor loss in *TCL1* leukemic mice triggers CLL toward a high-grade lymphoid malignancy. **A** FC^1^ analysis of CD19+ CD5+ cells in *TCL1* vs *Bcor*^*−/−*^*;TCL1* mice at different time-points in (i) PB^2^ at 0–-6 months (*N* = 17, 14), 6–12 months (*N* = 10, 10) and >12 months (*N* = 11, 9); (ii) spleen at 0-6 months (*N* = 17, 14), 6–12 months (*N* = 9, 9) and >12 months (*N* = 11, 12); (iii) BM^3^ at 0-6 months (*N* = 17, 14), 6–12 months (*N* = 9, 9) and >12 months (*N* = 11, 12); Mean ± SD^4^. *P* values are indicated above each graph according to Mann-Whitney U test. (B) Representative images of H&E^5^ staining of spleen magnification 20× (*N* = 2 for each genotype; left panels) and 40× (*N* = 3 for each genotype; right panels): sections from *TCL1* (upper panels) and *Bcor*^*−/−*^*;TCL1* (bottom panels) mice. UPlanApo 40×/0.85 NA objective, Olympus BX-51 microscope. (C) OS^6^ curve of *Bcor*^*−/−*^*;TCL1* (*N* = 25) compared to *Bcor*^*+/+*^ (*N* = 19), *Bcor*^*−/−*^(*N* = 25) and *TCL1* (*N* = 20) controls. Survival curves are compared using a Long-rank (Mantel-Cox) test. ^1^Flow cytometry; ^2^Peripheral Blood; ^3^Bone Marrow; ^4^Standard Deviation; ^5^Hematoxylin and Eosin; ^6^Overall survival.

To explore the impact of *Bcor* loss in CLL, we bred *Bcor*^*−/−*^ with Eµ*-TCL1 (TCL1)* mice (supplementary Fig. 5A). As shown in Fig. 1A, we found a higher number of CD19+ CD5+ B cells in *Bcor*^*−/−*^*;TCL1* compared to *TCL1* mice at different time-points of age in PB (0–6 months: 582 cells/mL ± 477.8 *vs* 305.7 cells/mL ± 242.2, respectively; 6–12 months: 9315 cells/mL ± 17250 *vs* 5324 cells/mL ± 6174, respectively; >12 months: 14665 cells/mL ± 15639 *vs* 12290 cells/mL ±  16826, respectively), spleen (0–6 months: 8.59 cells × 10^6^ ± 3.42 *vs* 3.75 cells × 10^6^ ± 2.04; 6–12 months: 47.75 cells × 10^6^ ± 21.70 *vs* 25.75 cells × 10^6^ ± 18.19; >12 months: 79.68 cells × 10^6^ ± 52.38 *vs* 34.36 cells × 10^6^ ± 31.93) and BM (0–6 months: 2.02 cells × 10^6^ ± 1.16 *vs* 0.83 cells × 10^6^ ± 0.75; 6–12 months: 10.21 cells × 10^6^ ± 5.64 *vs* 4.30 cells × 10^6^ ± 2.69; >12 months: 14.38 cells × 10^6^ ± 18.46 *vs* 4.74 cells × 10^6^ ± 5.24). To have a proof of clonality of the lymphoproliferations highlighted by flow cytometry, we conducted an analysis of IGHV rearrangements in splenic sorted B cells, revealing monoclonality with a perfect match of 100% to the IGHV germline sequences in *Bcor*^*−/−*^*; TCL1* mice (supplementary Fig. 5B). Concomitant Bcor loss and TCL1 overexpression enhanced alterations of splenic Fo and BM recirculating B cells of *Bcor*^*−/−*^ mice (supplementary Fig. 5C). At necropsy, *Bcor*^*−/−*^*; TCL1* mice showed splenomegaly, due to a diffuse infiltration by medium-large size cells with scattered mitotic figures, completely disrupting organs’ architecture (Fig. 1B). This observation was confirmed in 15 analyzed *Bcor*^*−/−*^*; TCL1* mice. These cells were characterized by nuclei with dispersed chromatin, well-defined nucleoli and broad cytoplasm. *TCL1* mice presented a prevalence of small B lymphocytes with round nuclei with condensed chromatin and scant nucleoplasms. Survival of *Bcor*^*−/−*^*;TCL1* mice (median survival 334 days with a range of 30–361 and 28% of survival at day 365) was significantly reduced compared to *Bcor*^*−/−*^(median survival undefined and 100% of survival at day 365)*, TCL1* (median survival undefined, range 131–355 and 65% of survival at day 365) and *Bcor*^*+/+*^ mice (median survival undefined and 100% of survival at day 365) (Fig. 1C).

### Adoptive transfer of *Bcor*^*−/−*^*; TCL1* leukemic splenocytes induces a more rapidly lethal disease in mice

**Fig. 2:**
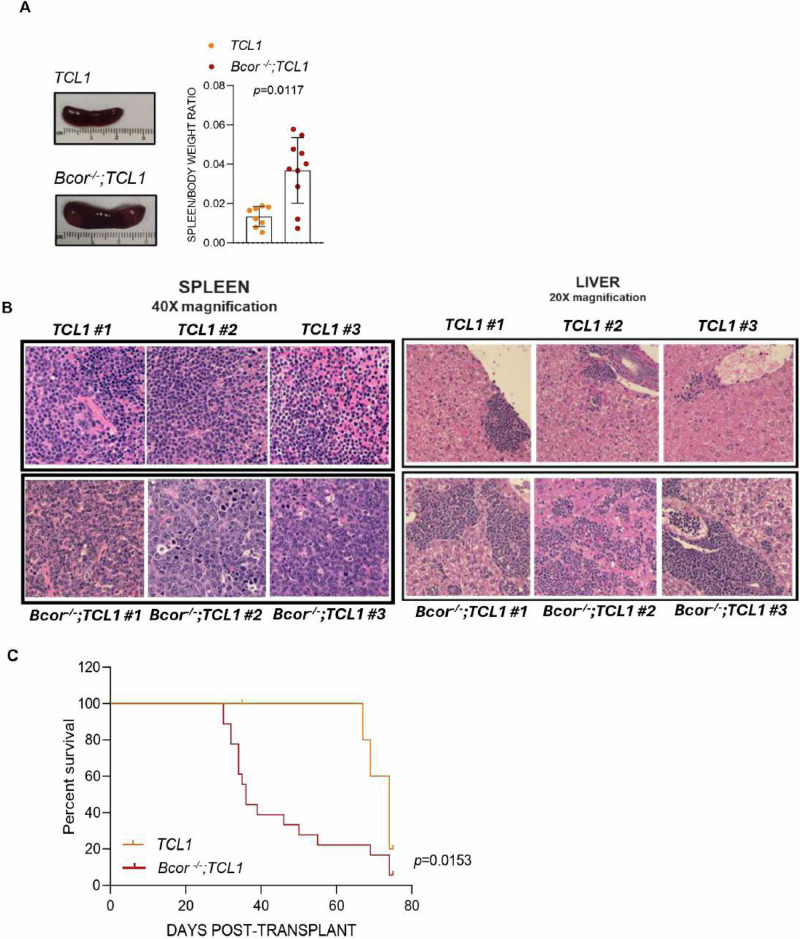
*Bcor*^*−/−*^*; TCL1* transplanted mice closely mimic RT found in humans phenotypically. (A) Representative images of spleen (left) and scatter dot plot graph showing spleen/body weight ratios (right) of *Bcor*^*−/−*^*; TCL1* (*N* = 10) compared to *TCL1* (*N* = 8) transplanted mice. *P* value is indicated above the graph according to Mann-Whitney U test. (B) Representative images of H&E^1^ staining of spleen (left; magnification 40×) and liver (right; magnification 20×) sections from *TCL1* (upper panels) and *Bcor*^*−/−*^*;TCL1* (bottom panels) transplanted mice (*N* = 3, respectively). UPlanApo 40×/0.85 NA objective, Olympus B×-51 microscope. (C) OS^2^ curves of *Bcor*^*−/−*^*;TCL1* (*N* = 18) compared to *TCL1* (*N* = 9) transplanted mice showing a median survival of 36 (range 30–69; 5.56% of survival at day 75) *vs* 74 (range 67–74; 20% of survival at day 75) days, respectively. Survival curves were compared using a Long-rank (Mantel-Cox) test. ^1^Hematoxylin and Eosin; ^2^Overall survival.

To promote a disease with accelerated kinetics we performed transplantation of splenocytes from diseased *Bcor*^*−/−*^*; TCL1* into C57BL/6 mice (supplementary Fig. 6A). In *Bcor*^*−/−*^*; TCL1* transplanted mice we found significant leukocytosis (WBC count: 14.71 ± 13.41 *vs* 4.18 ± 1.58 cells/µL), anemia (HGB values: 9.73 ± 4.09 *vs* 13.34 ± 0.64 g/dL) and thrombocytopenia (PLTs count: 209.7 × 10^3^ ± 113.9 *vs* 395.2 × 10^3^/µL ± 152.7) compared to *TCL1* transplanted mice (supplementary Fig. 6B). *Bcor*^*−/−*^*; TCL1* transplanted mice had significantly enlarged liver and spleen compared to *TCL1* transplanted mice (spleen/body weight ratios: 0.036 ± 0.016 *vs* 0.013 ± 0.005, respectively; Fig. 2A). Lymph nodes were not significantly involved in the neoplastic phenotype of *Bcor*^*−/−*^*; TCL1* mice. Histopathology of spleen and liver of transplanted mice was consistent with the non-transplanted counterpart in terms of infiltration by medium-large size cells mimicking RT (Fig. 2B). *Bcor*^*−/−*^*; TCL1* transplanted mice showed higher levels of CD19+ CD5+ cells compared to *TCL1* transplanted mice in spleen (275.3 × 10^6^ cells ± 172.3 vs 53.13 × 10^6^ cells ±  97.80, respectively), BM (8.68 cells × 10^6^ ± 8.52 vs 1.10 cells × 10^6^ ± 0.99, respectively) and liver (61.14% ± 29.84 vs 4.85% ± 3.69, respectively) (supplementary Fig. 6C). Clonality of splenic CD19+ CD5+ cells was also confirmed in *Bcor*^*−/−*^*; TCL1* transplanted mice (supplementary Fig. 6D). Additionally, neoplastic cells of *Bcor*^*−/−*^*;TCL1* transplanted mice presented significant increased Ki-67 levels compared to *TCL1* mutant in spleen (19.4% ± 6.44 *vs* 10.7% ± 3.2, respectively) and liver (47.24% ± 27.05 *vs* 13.15% ± 4.64, respectively) (supplementary Fig. 6E), suggesting the transformation of *TCL1* CLLs towards a high-proliferating disease reminiscent of RT. Survival of *Bcor*^*−/−*^*;TCL1* transplanted mice was significantly reduced (median survival of 36, range 30–69; 5.56% of survival at day 75) compared to *TCL1* transplanted mice (median survival of 74, range 67–74; 20% of survival at day 75) (Fig. 2C). These data indicated that *Bcor*^*−/−*^*; TCL1* mice mimic both clinical and phenotypic features of human RT.

### Splenic B cells of *Bcor*^*−/−*^*; TCL1* mice exhibit a transcriptional signature reminiscent of human RT

**Fig. 3:**
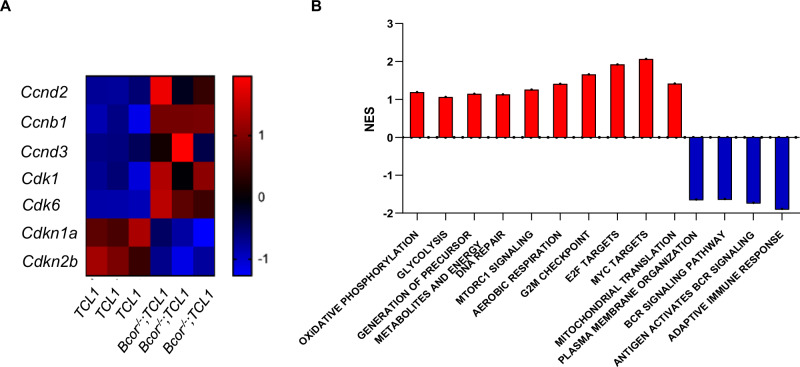
Transcriptomic profile *of Bcor*^*−/−*^*; TCL1* mice mimic human RT. **A** Heatmap of selected DEGs^1^ (FDR^2^ *q* < 0.05) showing upregulation of *Ccnd2* (log2FoldChange = 1.887; *padj* < 0.0001), *Ccnb1* (log2FC = 1.505; *padj* < 0.0001), *Ccnd3* (log2FC = 1.546; *padj* < 0.0029), *Cdk1* (log2FC = 1.328; *padj* = 0.0001), *Cdk6* (log2FC = 2.324; *padj* < 0.0001), and downregulation of *Cdkn1a* (log2FC = −1.885; *padj* = 0.0003), *Cdkn2b* (log2FC = −1.621; *padj* < 0.0001) in splenic B cells sorted from *Bcor*^*−/−*^*;TCL1* compared to *TCL1* mice (*N* = 3, both). **B** Bar graph showing NES^3^ of deregulated pathways (“Oxidative Phosphorylation” NES = 1.19; “Glycolysis” NES = 1.06; “Generation of precursor metabolites and energy” NES = 1.15; “DNA Damage Repair” NES = 1.13; “mTORC1 Signalling” NES = 1.26; “Aerobic Respiration” NES = 1.41; “G2M_Checkpoint” NES = 1.66; “E2F Targets” NES = 1.92; “MYC_Targets” NES = 2.07; “Plasma Membrane Organization” NES = −1.64; “BCR Signaling Pathway” NES = −1.63; “Antigen Activates BCR Signaling” NES = −1.73; “Adaptive_Immune_System” NES = −1.89 “Mitochondrial_Translation” NES = 1.42) resulted from pathway enrichment analysis in splenic sorted B cells from *Bcor*^*−/−*^*;TCL1* compared to *TCL1* mice (*N* = 3, both) after RNA-seq analysis. ^1^Differential Expressed Genes; ^2^False Discovery Rate; ^3^Normalized Enriched Score.

To dissect the molecular mechanisms underlying CLL-to-RT transition, we performed RNA-seq in transplanted splenic B cells identifying 1338 upregulated and 1674 downregulated genes in *Bcor*^*−/−*^*; TCL1* compared to *TCL1* mice (*N* = 3 for all genotypes) (supplementary Table 1). Enrichment analysis showed that *Bcor* deletion cooperates with *TCL1* to activate transcriptional programs sustaining cell proliferation, cell cycle progression and contrasting apoptosis. This signature included the upregulation of cyclins (*Ccnd2*, *Ccnb1*, *Ccnd3*) and cyclin dependent kinases (*Cdk1*, *Cdk6*), and the downregulation of cyclin dependent kinase inhibitors (*Cdkn1a*, *Cdkn2b*) (Fig. 3A). Furthermore, gene set analysis revealed upregulation of oncogenic and metabolic processes in association with downregulation of immunological pathways, like oxidative phosphorylation (OXPHOS) and DNA Damage Repair (DDR) processes (Fig. 3B). These data indicated that *Bcor*^*−/−*^*; TCL1* mice closely mimic RT found in humans at the transcriptional level [1, 14, 15].

To further validate the role of *BCOR* deregulations in RT patients, we analysed metadata from three recent studies [1, 14, 15] comparing longitudinal samples from CLL patients who developed RT. At genomic level, whole-genome sequencing and whole-exome sequencing did not identify mutations in the *BCOR* gene within the bulk tumor populations of RT samples. Conversely, the integration of single-cell DNA sequencing (scDNA-seq), copy number alteration analysis, and RNA-seq data led to the identification of *BCOR* gene mutations, loss of heterozygosity (LOH) within the *BCOR* locus, and transcriptomic deregulations of *BCOR* molecular partners involved in the Polycomb Repressive Complex (PRC) in 6 out of 19 RT patients. Specifically, the DNA sequencing at single-cell level, led to the identification of RT subclones carrying a *BCOR* mutation, in 1 out of 4 patients analysed (supplementary Fig. 7A). At the structural level, the copy number alteration analysis revealed loss of heterozygosity (LOH) at the locus on the chromosome X, where the *BCOR* gene is located, in 1 out of 19 RT patients analysed (supplementary Fig. 7A). This structural variation may be relevant to RT oncogenic processes, potentially reducing the tumor suppressor function of BCOR. At transcriptomic level, RNA-seq data from 6 patients with paired CLL and RT samples revealed downregulation of genes encoding several molecular partners of BCOR within the PRC in 6 out of 6 RT patients (supplementary Fig. 7B). The PRC is a multiprotein complex involved in the transcriptional repression of target genes through histone modifications. Proteins within the PRC include BCOR, EZH1, LCOR, CBX4, CBX7, PHC1, PHC3, JARID2 and USP7 (supplementary Fig. 7C). Additionally, only male patients exhibited direct alterations in the *BCOR* gene, including subclonal mutations or loss of heterozygosity (LOH), while females (*n* = 3) displayed impaired transcriptomic profiles associated with PRC genes.

### *Bcor*^*−/−*^*; TCL1* RT mice exhibit alterations of the T cell compartment

**Fig. 4:**
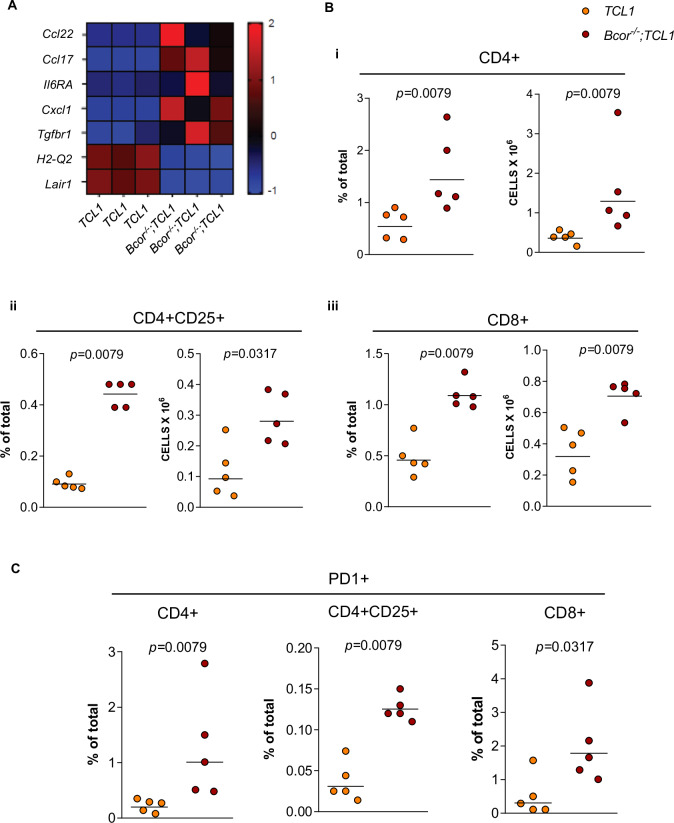
In vivo effects of Bcor deletion and TCL1 overexpression in neoplastic B cells and T cells from TME. **A** Heatmap of selected DEGs^1^ (FDR^2^ < 0.05, *p* adj^3^ < 0.05 and log Fold Change threshold of 1) showing upregulation of *Ccl17* (log2FC = 5.71; *padj* < 0.0001), *Ccl22* (log2FC = 7.73; *padj* < 0.0001), *Il6ra* (log2FC = 1.36; *padj* = 0.001), *Cx3cl1* (log2FC = 6.86; *padj* < 0.0001), *Tgfbr1* (log2FC = 1.04; *padj* = 0.003) and dowregulation of *H2-Q2* (log2FC = −3.50; *padj* < 0.0001) and *Lair1* (log2FC = −5.26; *padj* < 0.0001) in splenic-sorted B cells from *Bcor*^*−/−*^*;TCL1* compared to *TCL1* mice (*N* = 3, both). **B** FC^4^ analysis of the BM^5^ T-cell compartment of *TCL1 vs Bcor*^*−/−*^*; TCL1* mice showing altered (i) CD4+ (*N* = 5), (ii) CD4 + CD25+ (*N* = 5) and (iii) CD8+ (*N* = 5) cells frequencies and relative CD4+ (*N* = 5), CD4 + CD25+ (*N* = 5) and CD8+ (*N* = 5) cell number. Mean ± SD^6^. *P* values are indicated above each graph according to Mann-Whitney U test. **C** FC^4^ analysis of *TCL1 vs Bcor*^*−/−*^*; TCL1* mice BM^5^ samples showing the frequencies of the PD-1 marker gated on CD4+ (*N* = 5), CD4 + CD25 + (*N* = 5), and CD8+ (*N* = 5) cell populations. Mean ± SD^6^. *P* values are indicated above each graph according to Mann-Whitney U test. ^1^Differential Expressed Genes; ^2^False Discovery Rate; ^3^Adjusted *p*-value; ^4^Flow Cytometry; ^5^Bone Marrow; ^6^Standard Deviation.

To further explore the biological impact of Bcor loss in B cells of the *TCL1* model, we analyzed the tumor microenvironment of *Bcor*^*−/−*^*; TCL1* mice. Pathway enrichment analysis performed with Reactome and KEGG databases revealed an impaired immune-microenvironment regulation in transplanted *Bcor*^*−/−*^*; TCL1* compared to *TCL1* mice. Specifically, *Bcor*^*−/−*^*; TCL1* malignant cells showed upregulation of *Ccl17* and *Ccl22* chemokines that attract regulatory T (Treg) and Th2 cells in tumor microenvironment (TME). We also found overexpression in genes involved in microenvironment-dependent growth of neoplastic B cells, such as *Il6ra*, *Cx3cl1* and *Tgfbr1*. Conversely, *Bcor*^*−/−*^*; TCL1* samples exhibited downregulation of *H2-Q2* and *Lair1* genes, involved in adaptive immune system and regulating the BCR activation induced by TME interactions (Fig. 4A). These data suggested that RT developed in *Bcor*^*−/−*^*; TCL1* mice involved a deregulation of the local immune response. Based on this observation, we analyzed the T-cell compartment within the BM ecosystem of *Bcor*^*−/−*^*;TCL1* demonstrating a significant 2-, 2.67- and 1.86- fold increase of CD4+ (1.55 × 10^6^ ± 1.15), CD4 + CD25+ (0.29 × 10^6^ ± 0.08) and CD8+ (0.71 × 10^6^ ± 0.10) cells compared to *TCL1* mice (0.39 × 10^6^ ± 0.15 vs 0.11 × 10^6^ ± 0.09 vs 0.35 × 10^6^ ± 0.15, respectively; Fig. 4B). *Bcor*^*−/−*^*; TCL1* mice showed a significantly higher expression of PD-1 compared to *TCL1* mice in CD4+ (1.26% ± 0.95 vs 0.22% ± 0.11, respectively), CD4 + CD25+ (0.13% ± 0.01 vs 0.04% ± 0.02) and CD8+ cells (2.00% ± 1.14 vs 0.51% ±  0.61; Fig. 4C). Our results indicated that an impaired T cells function was associated with the RT phenotype of *Bcor*^*−/−*^*; TCL1* mice with potential pathogenic implications.

### Neoplastic B cells of *Bcor*^*−/−*^*; TCL1* RT mice exhibit Notch1 signaling activation

**Fig. 5:**
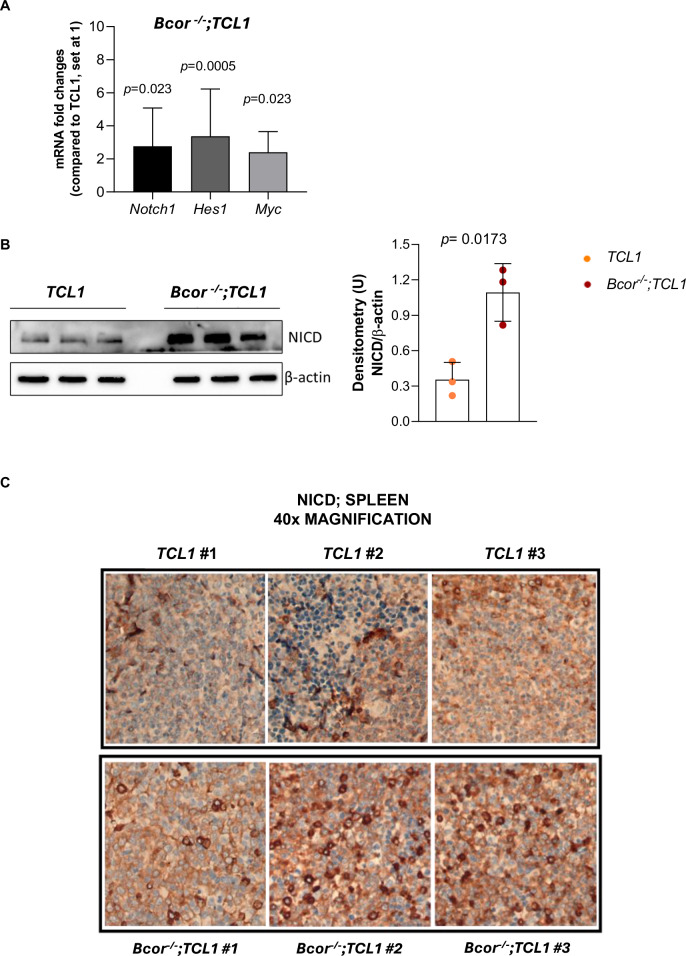
*Bcor*^*−/−*^*; TCL1* mice exhibit Notch1 signalling activation. **A** Real-time qPCR analysis of *Notch1*, *Hes1* and *Myc* mRNA levels in splenic flow sorted B cells from *TCL1* and *Bcor*^*−/−*^*; TCL1* transplanted mice (*N* = 5, both). Mean ± SD^1^. *P* values are indicated above each graph according to Mann-Whitney U test. **B** Representative WB^2^ (left) analysis and relative densitometry graphs (right) of NICD^3^ in splenic flow sorted B cells from *TCL1* and *Bcor*^*−/−*^*; TCL1* transplanted mice. Densitometry analyses are normalized to β-Actin and performed using Image Lab software. **C** Representative IHC^4^ staining showing NICD^3^- positive cells on splenic sections from *TCL1* and *Bcor*^*−/−*^*; TCL1* transplanted mice (*N* = 3, both). Magnification 40× (UPlanApo 40×/0.85 NA objective, Olympus BX-51 microscope). ^1^Standard Deviation; ^2^Western Blot; ^3^Notch1 Intracellular Domain: ^4^Immunohystochemestry.

*NOTCH1* mutation has been described as a driving oncogenic event associated with high-risk CLL and RT onset. *NOTCH1* mutation prevents its proteasomal degradation thus enhancing its own activation and transcription of some pro-survival genes [1–7]. Based on this, we investigated the involvement of Notch1 signaling activation in the disease of *Bcor*^*−/−*^*; TCL1* mice. RT-qPCR analyses of splenic B cells revealed a significant increase of the *Notch1* mRNA (2.76-fold increase) along with its downstream targets *Hes1* (3.36-fold increase) and *Myc* (2.40-fold increase) (Fig. 5A) while western blot data showed a significant increase of the activated Notch1 (3.11-fold increase) protein levels in *Bcor*^*−/−*^*; TCL1* compared to *TCL1* mice (Fig. 5B). IHC detection of the Notch1 Intracellular Domain (NICD), the active fragment of the NOTCH1 protein, showed a strong reactivity in nearly 100% RT large B cells infiltrating the spleen of *Bcor*^*−/−*^*; TCL1* mice compared to a less intense and more variable positivity in *TCL1* sections (Fig. 5C).

### Notch1-inhibition delayed CLL-to-RT transformation by affecting both neoplastic B cells and T cells in *Bcor*^*−/−*^*; TCL1* mice

**Fig. 6:**
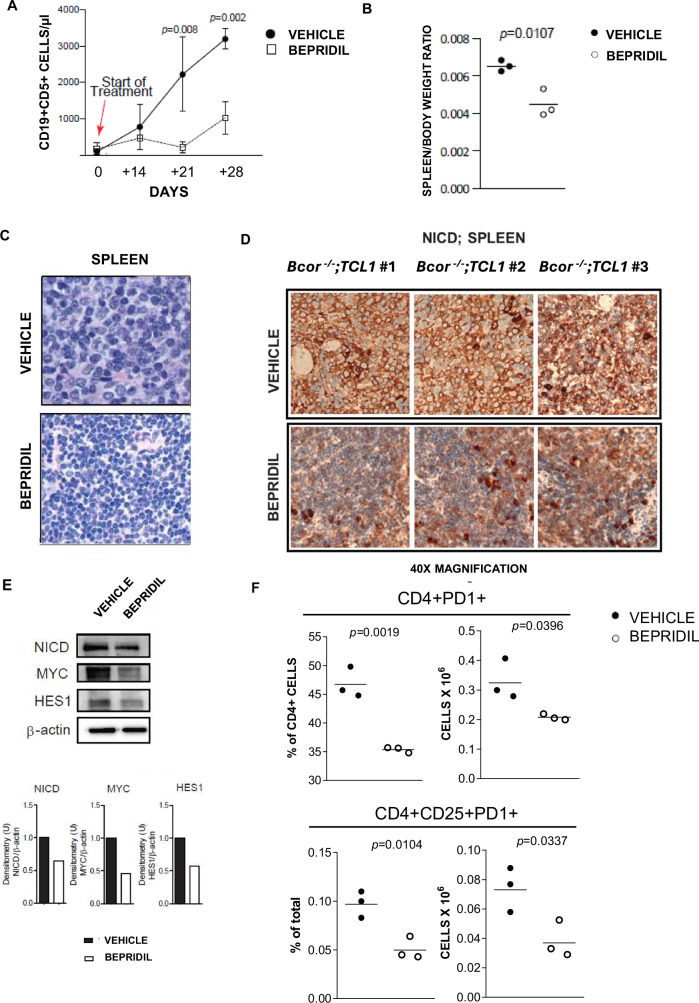
Cellular and molecular effects of bepridil treatment on *Bcor*^*−/−*^*; TCL1* mice outcome. **A** Curve graph showing the time course differences between vehicle (*N* = 3) *vs* bepridil-treated (*N* = 3) groups of *Bcor*^*−/−*^*; TCL1* transplanted mice in number of PB^1^ CD19+ CD5+ : day 0, 91.98 cells/mL ± 87.68 *vs* 163.3 cells/mL ± 172, respectively, *p* = 0.645; day 14, 772.5 cells/mL ± 625 *vs* 466.9 cells/mL ± 322.7, respectively, *p* = 0.629; day 21, 2,220 cells/mL ± 1021 *vs* 211.7 cells/mL ± 148.6, respectively, *p* = 0.008; day 28, 3195 cells/mL  ± 282 *vs* 1023 cells/mL ± 440, respectively, *p* = 0.002. Mean ± SD^2^. *P* values are calculated for each time-point according to U *t*-test and indicated above the graph in case of statistical significance. (B) Scatter dot plot graph showing the spleen/body weight ratios of transplanted *Bcor*^*−/−*^*; TCL1* mice after treatment with vehicle *vs* bepridil (0.0065 ± 0.0003 *vs* 0.0045 ± 0.0007; *p* = 0.0107; *N* = 3, both). Mean ± SD^2^. *P* value is indicated above the graph according to U *t*-test. (C) Representative images of H&E^3^ staining ( ~ 2x digital magnification of the 40X magnification acquisitions) of splenic sections from *Bcor*^*−/−*^*; TCL1* transplanted mice treated with vehicle (upper panel, *N* = 3) or bepridil (bottom panel, *N* = 3). Images evidence a change in cell-morphology with a prevalence of small monomorphic CLL^4^ cells after bepridil treatment (bottom panel), reminding the leukemic cells of *TCL1* mice (shown in Fig. 1), in place of the large sized cells characterizing the RT^5^ phenotype of *Bcor*^*−/−*^*; TCL1*, which are present in the spleen of vehicle mice instead (upper panel). Magnification 40X (UPlanApo 40×/0.85 NA objective, Olympus BX-51 microscope). (D) Representative IHC^6^ staining on splenic sections from *Bcor*^*−/−*^*; TCL1* mice showing the variation in number of NICD^7^-positive cells between vehicle (upper panel; *N* = 3) and bepridil-treated (bottom panel; *N* = 3) groups. Magnification 40X (UPlanApo 40×/0.85 NA objective, Olympus BX-51 microscope). (E) Representative WB^8^ analysis (top position) and relative densitometry graphs (bottom position) showing the differences in NICD^7^, MYC and HES1 protein levels in splenic-sorted B cells from a *Bcor*^*−/−*^*; TCL1* transplanted mouse after vehicle (*N* = 3) vs bepridil (*N* = 3) treatment. Densitometry analyses are normalized to β-Actin and performed using Image Lab software. (F) FC^9^ analysis of the PD-1 marker in BM^10^ samples of *Bcor*^*−/−*^*;TCL1* transplanted mice treated with bepridil (*N* = 3) compared to the vehicle (*N* = 3), gated on CD4+ (46.77% ± 2.67 *vs* 35.37% ± 0.49, respectively, and absolute number 0.33 × 10^6^ ± 0.06 *vs* 0.21 × 10^6^ ± 0.01 cells, respectively; left) and CD4 + CD25+ (0.1% ± 0.013 *vs* 0.05% ± 0.012, respectively, and absolute number 0.07 × 10^6^ ± 0.007 *vs* 0.04 × 10^6^ ± 0.01 cells, respectively; right) T-cell populations. Mean ± SEM^11^. *P* values are indicated above the relative graphs according to U *t*-test. ^1^Peripheral Blood; ^2^Standard Deviation; ^3^Hematoxylin and Eosin; ^4^Chronic Lymphocytic Leukemia; ^5^Richter Transformation; ^6^Immunohystochemestry; ^7^Notch1 Intracellular Domain; ^8^Western Blot; ^9^Flow Cytometry; ^10^Bone Marrow; ^11^Standard Error of the Mean.

In order to modulate Notch1 overactivation, we treated RT mice with bepridil that we recently reported as anti-NOTCH1 molecule with anti-leukemic effects in CLL models [16, 17]. In vivo, bepridil treatment progressively reduced PB CD19 + CD5+ cells compared to the untreated mice (day 0: 91.98 cells/mL ± 87.68 *vs* 163.3 cells/mL ± 172, respectively; day 14: 772.5 cells/mL ± 625 *vs* 466.9 cells/mL ±  322.7, respectively; day 21: 2220 cells/mL ± 1,021 *vs* 211.7 cells/mL ± 148.6, respectively; day 28: 3195 cells/mL ± 282 *vs* 1023 cells/mL ± 440, respectively; Fig. 6A). At sacrifice, we observed a 1.46-fold reduction of spleen size (spleen/body weight ratios of 0.0045 ± 0.0007 of treated mice *vs* 0.0065 ± 0.0003 of control; Fig. 6B) associated with a reduced infiltration by RT large B cells and a prevalence of small monomorphic cells more similar to the histo-pathology of *TCL1* mice (Fig. 6C). IHC analysis of splenic B cells showed a reduction of 80% in NICD levels in treated mice compared to vehicle (Fig. 6D). WB analysis further showed a reduction of NICD (0.29-fold-reduction) leading to the downregulation of its targets Myc (0.54 fold-reduction) and Hes1 (0.38 fold-reduction) at the protein level in treated mice compared to vehicle (Fig. 6E). Furthermore, bepridil treatment negatively modulated the frequency of CD4+ PD-1+ (0.21 × 10^6^ ± 0.01 *vs* 0.33 × 10^6^ ± 0.06 cells of the control) and CD4+ CD25+ PD-1+ (0.05 × 10^6^ ± 0.012 *vs* 0.1 × 10^6^ ± 0.013 cells of the control) (Fig. 6F). Our data indicated that the inhibition of Notch1 impacted on the RT phenotype by acting on both neoplastic B cells and TME.

## Discussion

*BCOR* disruptive mutations have been detected in several hematological malignancies, including B cell lymphomas and CLL [6, 8]. In these conditions, *BCOR* mutations often cooperate with other genetic alterations to drive the progression of the disease. Based on the tumor-suppressor role of *BCOR* [9, 18], we investigated the impact of Bcor loss in the context of CLL evolution through the generation of an in vivo pre-clinical model. Our data indicated that Bcor deficiency triggered the CLL phenotype of Eµ*-TCL1* mice toward a more rapidly lethal malignancy characterized by an increased number of proliferating large-sized cells, reminiscent of RT in humans. In a recent study, Nadeu et al. [1] listed *BCOR* as a driver gene in CLL and B-cell lymphomas, suggesting a potential role for *BCOR* deregulations in RT onset. Moreover, a comprehensive genome-wide sequencing analysis defined a high frequency of *Bcor* mutations in murine B-cell lymphomas, demonstrating that genetic disruption of *Bcor* accelerates the MYC-driven lymphomagenesis in the Eµ*-Myc* mouse model [18], further supporting the tumor suppressor role of *Bcor* in RT mice. These observations provide further evidence of the reliability of our *Bcor*^*−/−*^*; TCL1* model in recapitulating a RT disease.

Transcriptomic analysis of splenic B cells from *Bcor*^*−/−*^*; TCL1* mice showed alterations of key regulators of cell cycle and apoptosis that are considered molecular hallmarks of human RT together with *TP53* and *NOTCH1* mutations, as well as *MYC* amplification [1, 7, 14, 15]. This data further corroborates the reliability of our RT model, even at the transcriptional level. In addition, the transcriptional landscape identified in *Bcor*^*−/−*^*; TCL1* mice included deregulated metabolic processes and immunological pathways such as OXPHOS upregulation and BCR signalling downregulation. These results were consistent with recent studies showing that RT cells have a notable shift in the transcriptional program that converges into the activation of the OXPHOS pathway and downregulation of BCR signaling, the latter potentially compensated by activating Toll-like, MYC and MAPK pathways [1, 15]. Metabolic alterations have been identified in CLL as a result of disturbed calcium homeostasis [19], which could affect OXPHOS. This is due to the pivotal role of calcium in regulating mitochondrial ATP production.

Neoplastic B cells of *Bcor*^*−/−*^*; TCL1* mice also showed enrichment for genes involved in the DDR pathway, described to be a dominant mechanism driving RT transformation. Previous RNA profiling showed that DDR pathway genes are differentially regulated in human RT compared with CLL, and cells harbouring deregulations in the DDR pathways demonstrate high clonal expansion probability [14]. In line with these observations, our data indicated that *Bcor*^*−/−*^*; TCL1* mice closely mimic RT found in humans at the transcriptional and phenotypic level. Moreover, the integration of multiomics analysis performed with paired CLL and RT human samples (1) revealed that RT patients presented alterations of *BCOR* and its molecular partners involved in the PRC. The PRC has been shown to play a key role in normal hematopoiesis, and its dysregulation was closely associated with the pathogenesis of hematological malignancies. Notably, the alterations in *BCOR* and PRC genes identified through the metadata align with gender-specific expectations in RT patients. Validation in human samples support the relevance of our RT murine model and unleashes the need for future studies on BCOR and its partners within the PRC in human RT pathogenesis.

It has been demonstrated that CLL progression depends on interactions between neoplastic cells and tumor infiltrating lymphocytes within the TME [20, 21]. Our model showed upregulation of chemokines attracting Treg and Th2 cells in the TME and overexpression in genes involved in microenvironment-dependent growth of neoplastic B cells [22–25]. Conversely, *Bcor*^*−/−*^*; TCL1* samples exhibited downregulation of genes involved in adaptive immune system and regulating the BCR activation induced by TME interactions [26, 27]. Based on this observation, we analyzed the T-cell compartment within the BM ecosystem demonstrating the increase of CD4 + , CD4 + CD25+ and CD8+ cells compared to *TCL1* mice. Wierz et al. [28] described the expansion of Tregs with an enhanced immunosuppressive phenotype within the TME of a pre-clinical CLL model obtained by performing adoptive transfer of splenocytes from diseased *TCL1* into C57BL/6 recipient mice. This model was characterized by activated T cells displaying exhaustion features, such as the overexpression of PD1, LAG3, TIM3, and CTLA4. Interestingly, Tregs from *Bcor*^*−/−*^*; TCL1* mice showed a significantly higher expression of PD-1 compared to *TCL1* mice. In this regard, it has been demonstrated that resistance to anti-PD1 drugs in vivo was correlated with the molecular silencing of *Bcor*, indicating a potential role of BCOR in immune checkpoint regulation [29]. Based on the role of the PD-1/PD-L1 pathway in tumor immune evasion [30], our results indicated an impaired T cells function in contributing to the RT phenotype in the context of Bcor deficiency.

A crosstalk between RT cells and TME has been recently described in Eμ*-TCL1* mice with Akt [31] overactivation. In this model, Akt orchestrated the development of RT via the induction of Notch1 signaling in B cells, fuelled by microenvironmental T cells. Splenic B cells from *Bcor*^*−/−*^*; TCL1* mice revealed enhanced Notch1 signaling demonstrated by a significant increase of both transcript and protein levels of Notch1 and mRNA of its downstream target *Hes1*, compared to *TCL1* mice. These results support a mechanistic association between Bcor function and NOTCH1 signalling in CLL progression toward RT.

Most *BCOR*-mutated CLL cases are found in co-occurrence with high-risk prognostic factors, such as *IGHV* unmutated, trisomy 12 and *NOTCH1* aberrations [6, 32]. Furthermore, *BCOR* has been described as a tumor suppressor gene with the ability to transactivate NOTCH in the development of T-ALL in mice [9]. Thus, we assumed that activated Notch1 could be responsible for disease exacerbation in *Bcor*^*−/−*^*; TCL1* mice by reprogramming the immunological niche within the CLL-RT continuum.

To investigate the role of NOTCH1 signalling activation in our model, we inhibited NOTCH1 signalling in RT mice using the calcium-channel modulator bepridil [16, 17]. Bepridil has been shown to inhibit NOTCH1 in CLL cells more specifically than gamma secretase inhibitors (GSI), whose effects also impact NOTCH2 [16]. Splenic B cells of treated mice showed reduced levels of active NOTCH1 and of its targets MYC and HES1, compared to vehicle protein levels. After treatment, these mice exhibited a change in cell-morphology with a prevalence of small monomorphic CLL cells which remind the leukemic cells of TCL1 mice, in place of the medium-large sized cells characterizing our mouse model. The driving oncogenic role of *NOTCH1* deregulations have been widely explored in CLL [1–7, 14–17, 21, 31–37], pointing out NOTCH1 as a potential mechanism in RT evolution. Several preclinical studies have explored the effects of NOTCH1 inhibition in CLL models, showing promising results in terms of suppressing CLL cell proliferation and inducing apoptosis [16, 17]. In CLL, NOTCH1 activation have been described as an alternative mechanism underlying acquired resistance to ibrutinib, independent of point mutations in the BTK binding sites [17]. In this context, the antileukemic effects of ibrutinib was associated with NOTCH1 downregulation [17], suggesting that NOTCH1 inhibition could overcome the selective pressure of target therapies in resistant clones that may undergo RT progression. Growing evidence have been also provided about the critical role of NOTCH1 in driving resistances to anti-CD20 antibodies and BCL2 inhibitors, providing a rationale for NOTCH1 targeting in high-risk CLL and RT patients [36]. Additionally, the identification of *NOTCH1* aberrations in hematopoietic stem cells of CLL patients highlighted the contribution of NOTCH1 in driving the clonal expansion of cancer cells [38], suggesting that NOTCH1 inhibition could potentially lead to CLL regression or contrasting RT progression. Inhibition of NOTCH1 was demonstrated to be effective also in other NOTCH1 dependent tumors [39] further supporting its oncogenic role.

In addition to direct antineoplastic effects, the inhibition of Notch1 induced by bepridil negatively modulated the frequency of CD4 + PD-1+ and CD4 + CD25 + PD-1+ cells in *Bcor*^*−/−;*^ *TCL1* mice. These results were consistent with previous findings describing the involvement of NOTCH1 in regulating T-cell responses and Tregs differentiation [40] as well as PD-1 expression [41]. These data suggested that NOTCH1 inhibition could represent an effective therapeutic option acting on both the neoplastic clone and the immune deregulations found in RT.

In conclusion our study provides the first evidence of the tumor suppressor role of Bcor coupled with Notch1 deregulation in a pre-clinical model of high-grade lymphoid malignancy mimicking human RT. Our model represents a robust system to study new pathogenic mechanisms and interrogate novel therapies in a challenging disease with an extremely dismal outcome.

## Supplementary information

Supplemental file

Dataset 1

## Data Availability

For original data, please contact paolo.sportoletti@unipg.it.
