# Supplementary material for: Bcor loss promotes Richter transformation of chronic lymphocytic leukemia associated with Notch1 activation in mice
Source: Leukemia. 2025 Mar 20;39(5):1157–68. doi: 10.1038/s41375-025-02557-y (PMC12055590; doi:10.1038/s41375-025-02557-y)
Supplement: Supplementary file 1 — Supplemental file [file 41375_2025_2557_MOESM1_ESM.docx]

# Bcor loss promotes Richter transformation of chronic lymphocytic leukemia associated with Notch1 activation in mice

Chiara Rompietti^1*^, Francesco Maria Adamo^1*^, Daniele Sorcini^1^, Filomena De Falco^1^, Arianna Stella^1^, Giovanni Martino^1^, Barbara Bigerna^1^, Erica Dorillo^1^, Estevão Carlos Silva Barcelos^1^, Angela Esposito^1^, Clelia Geraci^1^, Roberta Arcaleni^1^, Jessica Bordini^2^, Lydia Scarfò^2^, Emanuela Rosati^3^, Paolo Ghia^2^, Brunangelo Falini^1^ and Paolo Sportoletti^1^

*CR and FMA contributed equally as co-first authors

# Supplemental Material

Supplemental Methods

Supplemental Reference

Supplemental Figure 1 (with legend) Supplemental Figure 2 (with legend) Supplemental Figure 3 (with legend) Supplemental Figure 4 (with legend)

Supplemental Figure 5 (with legend)

Supplemental Figure 6 (with legend)

Supplemental Figure 7 (with legend)

Supplemental Table 1 (with legend)

# Supplemental Methods

**Mouse strains**

Mice were bred and housed by the “Service Center of Preclinical Research” of Perugia and Plaisant Srl of Castel Romano (Rome) animal house facilities. Mouse manipulations were performed in accordance with the European Community guidelines and to the protocol reviewed and approved by the Italian Health Ministry (authorization n° 971/2020- PR – n°253/2024-PR).

The conditional knock-out (KO) mouse model for BCOR^11^ was developed deleting exons from 8 to 10 of the murine *Bcor* gene, resulting in frameshift and premature stop codon in exon 11. Homozygous *Bcor^flox/flox^* female and hemizygous *Bcor^flox/Y^* male mice (background C57BL/6) have been crossed with *CD19-Cre* mice^12^ (background C57BL/6) to generate *Bcor^flox/flox^*;*Cre*^+^ and *Bcor^flox/Y^;Cre*^+^ mice, both indicated as *Bcor^-/-^* (supplemental Figure 3A). The expression of CD19-Cre is constitutive so after crossing the two strains we obtained excision of PGK- Neo cassette and specifically restricted the deletion of murine *Bcor* conditional allele in the B-cell compartment. To generate compound *Bcor^-/-^; TCL1* mutant animals, we crossed *Bcor^-/-^* *with* the CLL mouse model Eμ*-TCL1^13^* (background C57BL/6; supplemental Figure 4A).

# Cre recombination PCR analysis

To verify the occurrence of Cre-mediated excision event of the *Bcor* locus, we performed a standard PCR on gDNA samples of splenic flow-sorted B cells from *Bcor^-/-^* and *Bcor^+/+^* mice, as previously described11.

**Real-time qPCR analyses**

To verify the loss of *Bcor* transcript, total mRNA from splenic flow-sorted B cells was extracted with mRNeasy micro kit (Qiagen, Hilden, Germany), retrotranscribed to cDNA using the Superscript IV First-Strand Synthesis System (Thermo Fisher Scientific, Massachusetts, US). *Bcor* mRNA levels were evaluated by qPCR (Applied Biosystem 7300 Real time PCR system) using the following specific primers.

Primers’ sequences:

*mBcor* For 5’-AGGCTATGAGGAAGTGGTCT-3’

*mBcor* Rev 5’-CATCATGCAGAGGCCTGGTT-3’

They were designed on exons 10 and 11 respectively, of murine *Bcor* gene transcript (*Mus Musculus*). β-actin was used for normalization and ΔΔCt formula used to calculate mRNA fold changes. To investigate the involvement of Notch1 signaling we analysed the expression levels of murine *Notch1* transcript and of its targets *Hes1* and *Myc* in splenic flow-sorted B cells from our mice by qPCR using the following specific primers.

Primers’ sequences:

*mNotch1* For 5’-CCCTTGCTCTGCCTAACGC-3’

*mNotch1* Rev 5’-GGAGTCCTGGCATCGTTGG-3’

*mHes1* For 5’-CCAAGCTAGAGAAGGCAGACA-3’

*mHes1* Rev 5’-GTCACCTCGTTCATGCACTC-3’

*mMyc* For 5’-AGTGCTGCATGAGGAGACAC-3’

*mMyc* Rev 5’-GGTTTGCCTCTTCTCCACAG-3’

# Adoptive Transfer (AT) procedure

Wild-type recipient C57BL/6N mice (female animals of 12-16 weeks old) were sub-lethally irradiated (4.5 Gy) in the MultiRad 225 X-Ray Irradiation System. After 24h, recipients were transplanted with frozen 2-2.5 x 107 splenic cells derived from one original leukemic mouse donor of *TCL1* or *Bcor^-/-^; TCL1* strain (leukemic burden >50%) by intravenous injection (I.V.). We used mice at second round of transplantation. Experiments were performed in at least three replicates using at least three original mouse donors for each strain. B cells from *Bcor^-/-^* and *Bcor^+/+^*mice did not engrafted on C57BL/6N recipients, therefore they were not included in experiments of serial transplantation.

In the context of bepridil administration, 2-2.5 x 10^7^ splenic cells from three original leukemic mouse female donors of Bcor^-/-^; TCL1 strain (leukemic burden >50%) were injected by I.V. procedure into N=3 recipients for each treatment arm (group #1: DMSO/vehicle; group #2: bepridil). After the evaluation of an effective (≥5-10% PB CD19+CD5+ cells) and comparable engraftment among samples, only mice at second round of transplantation received the treatment and then have been analysed through repeated PB sampling until euthanasia, everything according to the dedicated section method reported below. Only female C57BL/6N recipients were used for all the AT experiments.

**IGHV rearrangement and mutational status analysis**

Genomic DNA was isolated from splenic B cells from *Bcor^-/-^; TCL1* and *TCL1* mice and was subjected to PCR reaction using primers that detect VDJ regions from immunoglobulin heavy chain genes, as previously described (supplemental reference 1). Combinations of forward (VHJ558, VH7183, VH52Q) and reverse (JH4 intron) primers were used. The resulting PCR products were loaded on 1,5% agarose gel and samples resulted positive for the VH families indicated above were analysed by Sanger sequencing, using an AB 3500 genetic analyzer. The electropherograms obtained were directly analysed to distinguish between a polyclonal and a monoclonal sample and sequences were also examined using IgBLAST (http: //www.ncbi.nlm.nih.gov/igblast/) and IMGT/V-QUEST (<http://www.imgt.org/IMGT_vquest/share/textes/>)

# Peripheral blood count

Mice were anesthetized with isoflurane (3-5%) and peripheral blood (PB) samples were collected by retroorbital bleeding in EDTA coated microvette tubes. Complete blood count was performed using a DxH520 hematology analyzer (Beckman Coulter).

# Flow cytometry and cell-sorting

PB samples were pre-lysed with red blood cells lysis solution before analyses. Bone marrow (BM) was collected by aseptically flushing femurs and tibia while spleens and livers were cut into small pieces and crushed through FALCON cell strainers (40 µm; Corning Incorporated, New York, US). The resulting cells were re-suspended in PBS and incubated with the antibodies listed below. Cell acquisition was performed using BD FACS CANTO and BD FORTESSA (BD Biosciences). Data were analysed with FlowJo Vx.0.7 Flow Cytometry analysis software (Tree Star Inc, Ashland, OR). Disease onset and progression were defined by cell suspensions staining using with CLL murine markers against CD19 PE (Cat # 130-112-035, clone REA749, Miltenyi Biotec, Germany) and CD5 APC (Cat # 130-120-298, clone REA421, Miltenyi Biotec). The proliferation rate of leukemic cells was assessed by staining liver and splenic cells with intra-cellular anti-Ki67 PerCP-Cy5.5 (Cat # 561284, B56 (RUO), BD Biosciences, CA, US), gated on CD19+CD5+ cell population, after applying the protocol for cell membrane permeabilization using the FOXP3/Transcription, Factor Binding Buffer Set (Thermo Fisher Scientific/eBioscience).

Multicolor flow cytometry panels were used to analyse T- and B- cell compartments using the following antibodies:

-T-cell compartment, anti-mo CD3 APC (Cat # 100312, clone 145-2C11, BioLegend, San Diego, CA, US), anti-mo CD4 PerCP-Cy5.5 (Cat # 45-0042-82, clone eBioRM4-5, Thermo Fisher Scientific), anti-mo CD8 FITC (Cat # 14-0081-82, clone eBio 53-6.7, Thermo Fisher Scientific), anti-mo CD25 AlexaFluor 488 (Cat # 53-0252-82, clone eBio7D4, Thermo Fisher Scientific) and PD-1 PE-Cy7 (Cat # 135216, clone 29F.1A12, BioLegend)**.**

-B-cell compartment, anti-mo/hu B220 AlexaFluor 780 (Cat # 47-0452-82, clone RA3-6B2, Thermo Fisher Scientific), anti-mo IgM FITC (Cat # 11-5790-81, clone II/41, Thermo Fisher Scientific), anti-mo IgD PE- Cy7 (Cat # 25-5993-82, clone 11-26c(11-26), Thermo Fisher Scientific), anti-mo cKIT APC (Cat # 17-11-71-83, clone 2B8, Thermo Fisher Scientific), anti-mo CD43 PE (Cat # 12-0431-82, eBioR2/60, Thermo Fisher Scientific), anti- mo CD21 APC (Cat # 17-0212-82, clone eBio4E3 (4E3), Thermo Fisher Scientific) and anti-mo CD23 PE (Cat # 12-0232-82, clone B3B4, Thermo Fisher Scientific). BM B-cell subpopulations were defined as pre-pro-B-cells (B220low, CD43+, c-Kit+; pro-B cells B220low, CD43+, c-Kit-), pre-B cells (B220+, CD43-, IgM-, IgD-, c-Kit-; immature B cells B220+, CD43-, IgM+, IgD-, c-Kit-) and recirculating B cells (B220+, CD43-, IgM+, IgD+, c-Kit-). Splenic ~~cells~~ B-cell subpopulations were defined as follicular B cells (Fo; B220+, IgM+, IgD+, CD23+, CD21dim) and marginal zone B cells (MZ; B220+, IgM+, IgD-, CD23dim, CD21+). The gating strategies are described in supplemental Figure 1, 2.

Cell sorting of splenic CD19+ cells was performed by BD FACS-Aria III (BD Biosciences). All panels’ gates were drawn to exclude non-viable cells and debris and a purity >95% was verified to be achieved after each experiment.

# Histology and Immunohistochemistry

At sacrifice, mice were subjected to the autopsy procedure to harvest spleen, liver, and vertebral column. The latter was previously decalcified with neutral EDTA. All organs from euthanized mice were formalin fixed and paraffin embedded. Subsequently, 3 µm sections were cut and stained with hematoxylin and eosin (H&E) for morphological analyses. Immunohistochemistry on liver and splenic sections was performed with cleaved NOTCH1 rabbit mAb antibody (Val1744; Cat# 4147, clone D3B8, RRID: AB_2153348) from Cell Signaling Technology (Danvers, MA, USA), on a BenchMark ULTRA system (Roche Tissue Diagnostics, Oro Valley, AZ, USA), using the ultraView Universal Alkaline Phosphatase Red Detection Kit (Roche Tissue Diagnostics) and the ultraView Universal DAB Detection Kit (Roche Tissue Diagnostics), following manufacturer's instructions. Images were acquired at different magnifications using an Olympus BX-51 microscope (Olympus Life Science, Center Valley, Pennsylvania) with a UPlanApo 40×/0.85 NA objective.

# Western blot

Whole-cell lysates were extracted in RIPA buffer. Equal amounts of proteins were separated by 7.5 to 15% SDS-PAGE and transferred to nitrocellulose membranes. Blots, after blocking, were incubated with following primary antibodies to: BCOR rabbit polyAb (12107-1-AP) from ProteinTech Group Inc (Rosemont, IL, USA), cleaved NOTCH1 rabbit mAb (Val1744; Cat# 4147, clone D3B8, RRID:AB_2153348) from Cell Signaling Technology, HES1 (clone E-5, Cat #sc-166410) and MYC (clone 9E10, Cat # sc-40) mouse mAbs from Santa Cruz Biotechnology (Santa Cruz, CA, USA), β-actin Human/Mouse mAb (clone AC-15, Cat # NB 600-501) from Novus Biologicals LLC/Bio-Techne SRL (Milano, Italy).

Primary antibodies were detected using horseradish peroxidase-linked secondary antibodies (Jackson ImmunoResearch Laboratories) together with the SuperSignal™ West Pico PLUS Chemiluminescent Substrate (Thermo Fisher Scientific). Densitometric analyses was performed using Image Lab software (Bio-Rad).

# RNA sequencing

Total RNA was extracted from splenic flow-sorted B cells (CD19+) of *TCL1* and *Bcor^-/-^; TCL1* transplanted mice, using RNeasy Plus Kits (Qiagen). RNA samples (RNA Quality Number: 7-10; RQN=10 represents intact total RNA in a scale from 1 to 10) were characterized at transcriptional level by Standard RNA sequencing. Unique Differentially Expressed Genes (DEGs) identified (FDR < 0.05, *p* adj < 0.05 and log Fold Change threshold of 1; supplemental Table 1) were analysed using the integrated data analysis pipeline on Gene Set Enrichment Analysis (GSEA) software and used in enrichment analysis on Kyoto Encyclopedia of Genes and Genomes (KEGG) and Reactome pathway databases.

# Drug administration

The achievement of ~10% CD19+CD5+ cell population in PB was considered the starting point of treatment (day 0). Mice have been injected intraperitoneally with DMSO (vehicle) or bepridil (5 mg/kg) once daily, 5 days/week, over a 1-month period, as described before16. Disease trend has been evaluated by PB analyses (blood count and CD19/CD5 flow cytometry) at day 0, +14, +21, +28, and mice were euthanized at the end of treatment for further analyses.

# Statistical analyses

Statistical analyses were performed with GraphPad v8 (GraphPad Software Inc.). For survival studies, we used the method of Kaplan-Meier and statistical significance of the overall survival (OS) was calculated using the log-rank (Mantel-Cox) test. Statistical differences between mean values were evaluated using unpaired nonparametric Mann-Whitney or, in case of small groups to analyze, unpaired parametric student’s *t*-test. Results were considered statistically significant with *P* value < 0.05. Statistical significance and groups’ size are indicated in the figure and supplemental figure legends

**Supplemental references**

1. Christopher J. Jolly, Norman Klix1 and Michael S. Neuberger. Rapid methods for the analysis of immunoglobulin gene hypermutation: application to transgenic and gene targeted mice. Nucleic Acids Research, 1997, Vol. 25, No. 10 1913–1919

Data Sharing Statement

For original data, please contact [paolo.sportoletti@unipg.it](mailto:paolo.sportoletti@unipg.it)


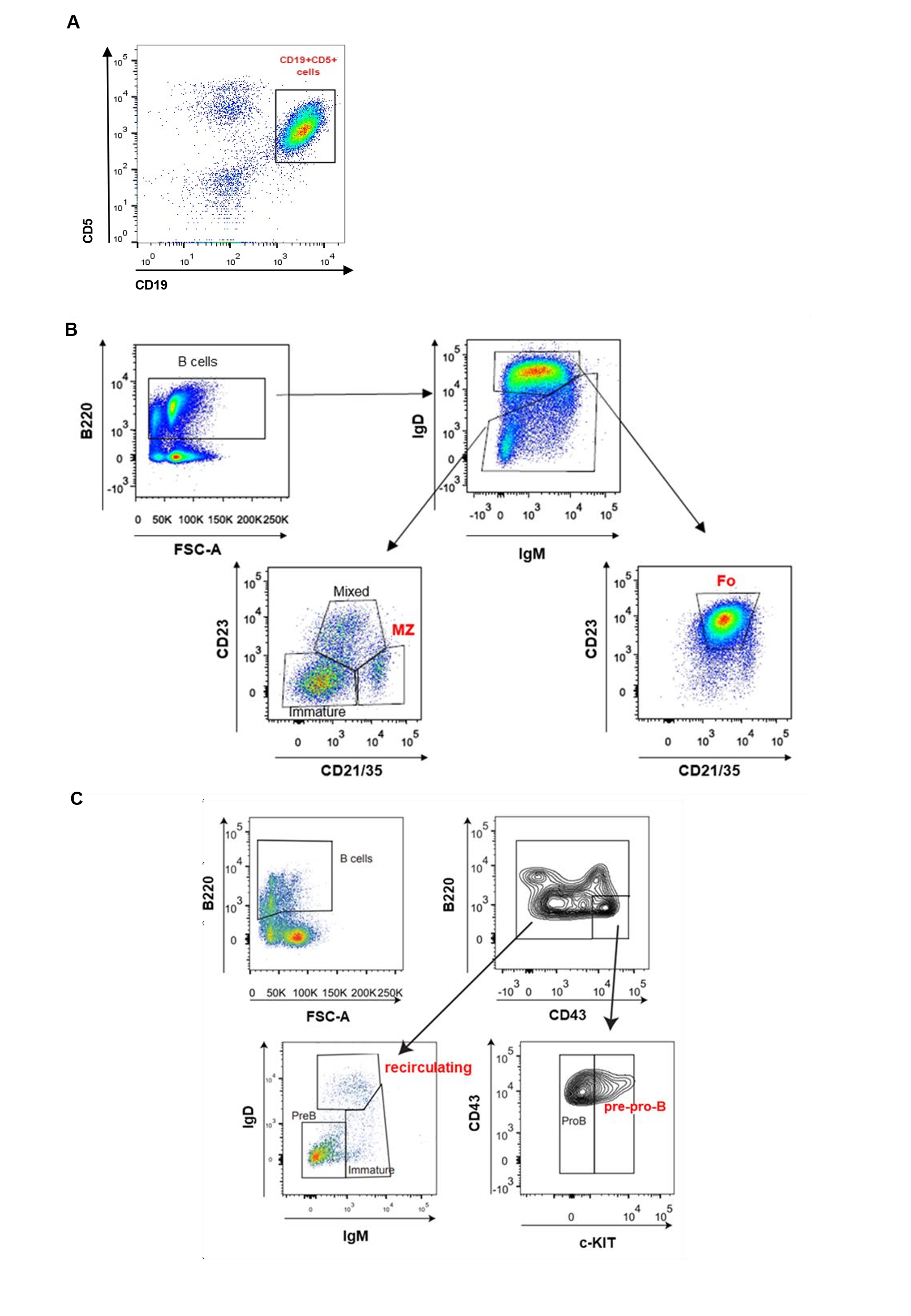


**Supplemental Figure 1**

(A) FC^1^ gating strategy designed to detect CD19+CD5+ cells. Antibodies fluorochromes used: CD19 PE and CD5 APC. (B) Representative FC^1^ gating strategy designed to discriminate splenic B-cell subpopulations in mice, including immature, mixed, MZ^2^ and Fo^3^ B cells. Antibodies fluorochromes used: B220 AlexaFluor780, IgM FITC, IgD PE-Cy7, CD21 APC and CD23 PE. (C) Representative FC^1^ gating strategy designed to discriminate BM^4^ B-cell subpopulations in mice, including pre-pro-, pro-, pre-, immature and recirculating mature B cells. Antibodies fluorochromes used: B220 AlexaFluor780, IgM FITC, IgD PE-Cy7, c-KIT APC and CD43 PE.

^1^Flow Cytometry; ^2^Marginal Zone; ^3^Follicular; ^4^Bone Marrow


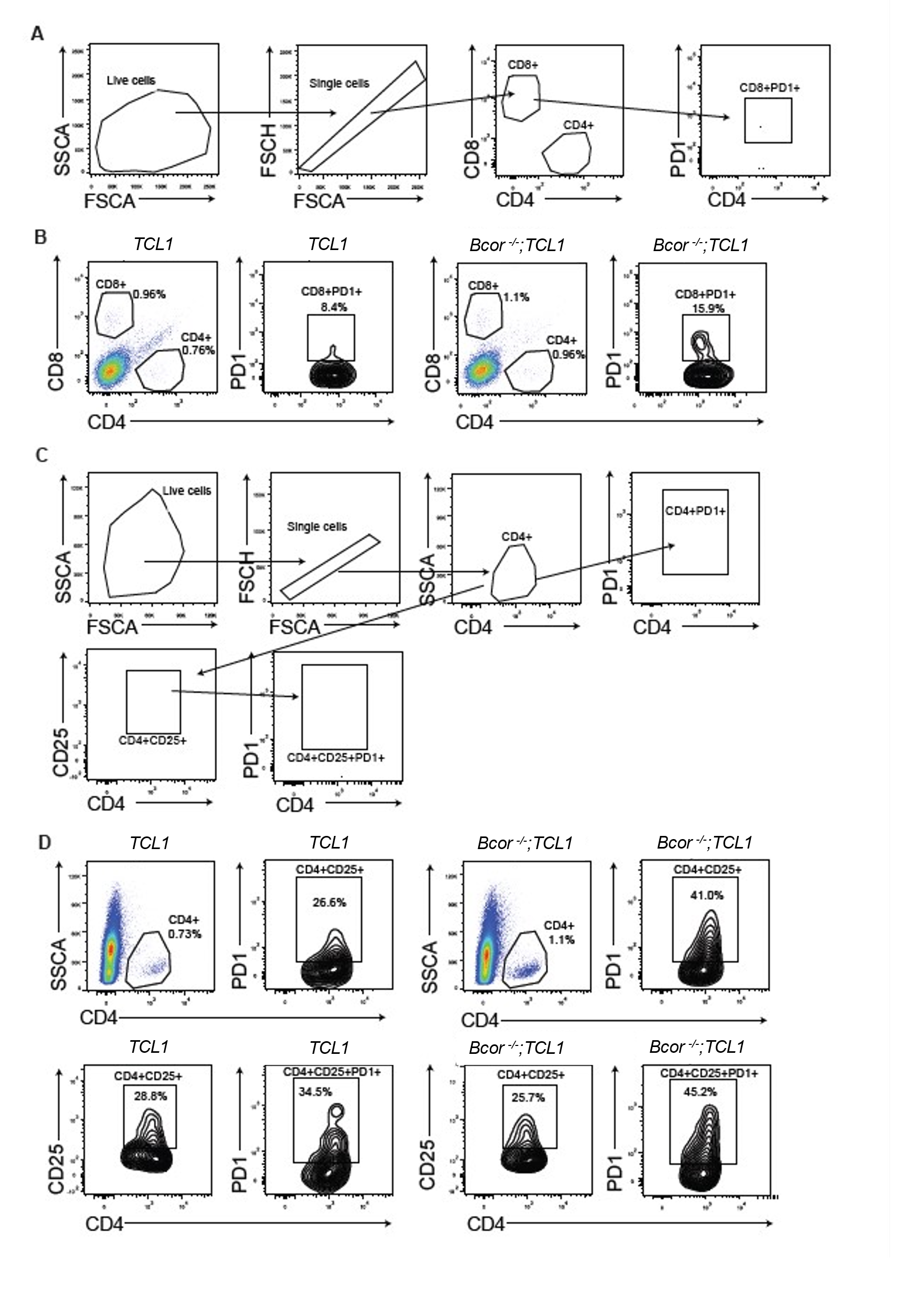


**Supplemental Figure 2.**

(A) FC^1^ gating strategy designed to discriminate murine CD8+PD-1+ T cells in the BM^2^ of *Bcor^-/-^; TCL1* and *TCL1* mice, and (B) relative representative result comparing the two mouse strains. Antibodies fluorochromes used: anti-mo CD4 PerCP-Cy5.5, anti-mo CD8 FITC, PD-1 PE-Cy7. (C) FC^1^ gating strategy designed to discriminate murine CD4+PD-1+ T cells and CD4+CD25+ T reg subpopulation in the BM^2^ of *Bcor^-/-^; TCL1* and *TCL1* mice, and (D) relative representative result comparing the two mouse strains. Antibodies fluorochromes used: anti-mo CD8 FITC, anti-mo CD4 PerCP-Cy5.5, anti-mo CD25 AlexaFluor 488, PD-1 PE-Cy7.

^1^Flow Cytometry; ^2^Bone Marrow


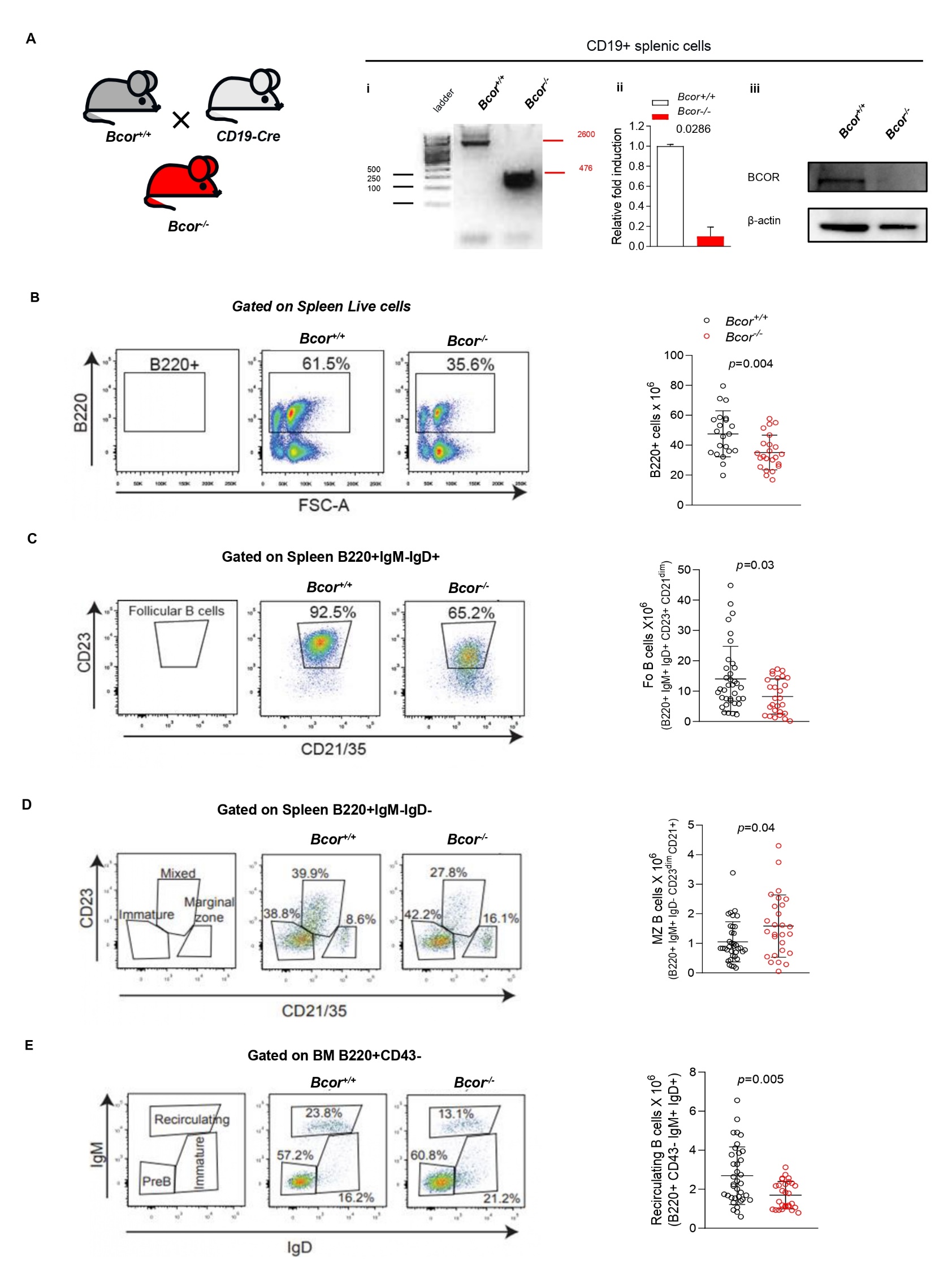
 **Supplemental Figure 3.**

(A) Left panel describes the mouse crossing strategy to generate murine genotypes used in this work. Right panel shows: i) representative result of the *Bcor* genomic locus recombination at the gDNA level in splenic sorted B cells (CD19+) from Bcor^+/+^ and Bcor^−/−^ mice (non-recombined *Bcor* locus= 2600 bp; Cre excised fragment= 476 bp); ii) column graph of *Bcor* mRNA levels analysed by real-time qPCR in splenic sorted B cells from Bcor^+/+^ and Bcor^−/−^ mice. *P* value is indicated above the graph according to Mann-Whitney U test; iii) representative WB^1^ analysis (Bcor molecular weight=186 kDa) of the Bcor protein in splenic sorted B cells from Bcor+/+ and Bcor−/−mice). (B). Representative result of FC^2^ dot plots (B220+; left) and scatter dot plot graph (right) showing the differences in number of splenic B220+ cells from *Bcor^−/−^*(N=23) mice compared to *Bcor^+^*^/+^ (N=21) controls. (C) Representative result of FC^2^ dot plots (B220, IgM, IgD, CD21, CD23; left) and scatter dot plot graph (right) showing the differences in number of splenic Fo^3^ B-cell population from *Bcor^−/−^* (N=27) mice compared to *Bcor^+^*^/+^ (N=36) controls. (D) Representative result of FC^2^ dot plots (B220, IgM, IgD, CD21, CD23; left) and scatter dot plot graph (right) showing the differences in number of MZ^4^ B-cell population from *Bcor^−/−^* (N=27) mice compared to *Bcor^+^*^/+^ (N=36) controls. (E) Representative result of FC^2^ dot plots (B220, IgM, IgD, c-KIT, CD43; left) and scatter dot plot graph (right) showing the differences in number of recirculating BM^5^ B cells from *Bcor^−/−^* (N=28) mice compared to *Bcor^+/+^* (N=36) controls. Mean ± SD^6^. *P* values are indicated above the graphs according to Mann-Whitney U test.

^1^Western Blot; ^2^Flow Cytometry; ^3^Follicular; ^4^Marginal Zone; ^5^Bone Marrow; ^6^Standard Deviation


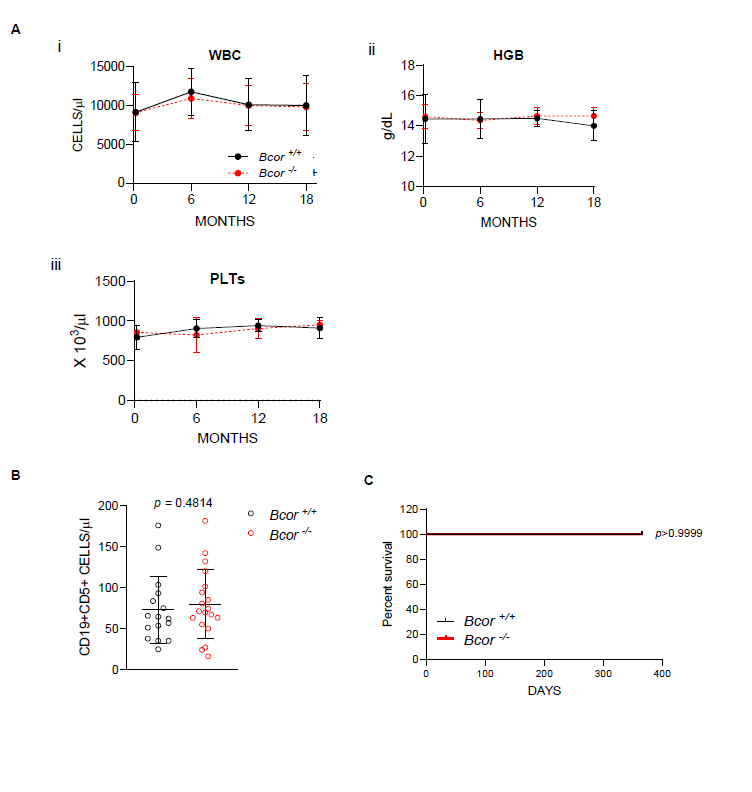


**Supplemental Figure 4**

(A) Curve graphs showing the time course differences in PB^1^ cell counts between *Bcor^-/-^* and *Bcor^+/+^* mice. i) WBC, ii) HGB^3^ and iii) PLTs^4^. (B) Scatter dot plot showing the number of PB CD19+CD5+ cells from *Bcor^+/+^* (N=16) and *Bcor^-/-^* (N=19) mice analysed by FC^5^. Mean ± SD^6^. *P* value is indicated above the graph according to Mann-Whitney U test. (C) OS^7^ curve of *Bcor*^-/-^ mice (N=25) compared to *Bcor^+/+^* controls (N=19). Survival curves are compared using a Long-rank (Mantel-Cox) test.

^1^Peripheral Blood; ^2^White Blood Cells; ^3^Hemoglobin; ^4^Platelets; ^5^Flow Cytometry; ^6^Standard Deviation; ^7^Overall Survival

**
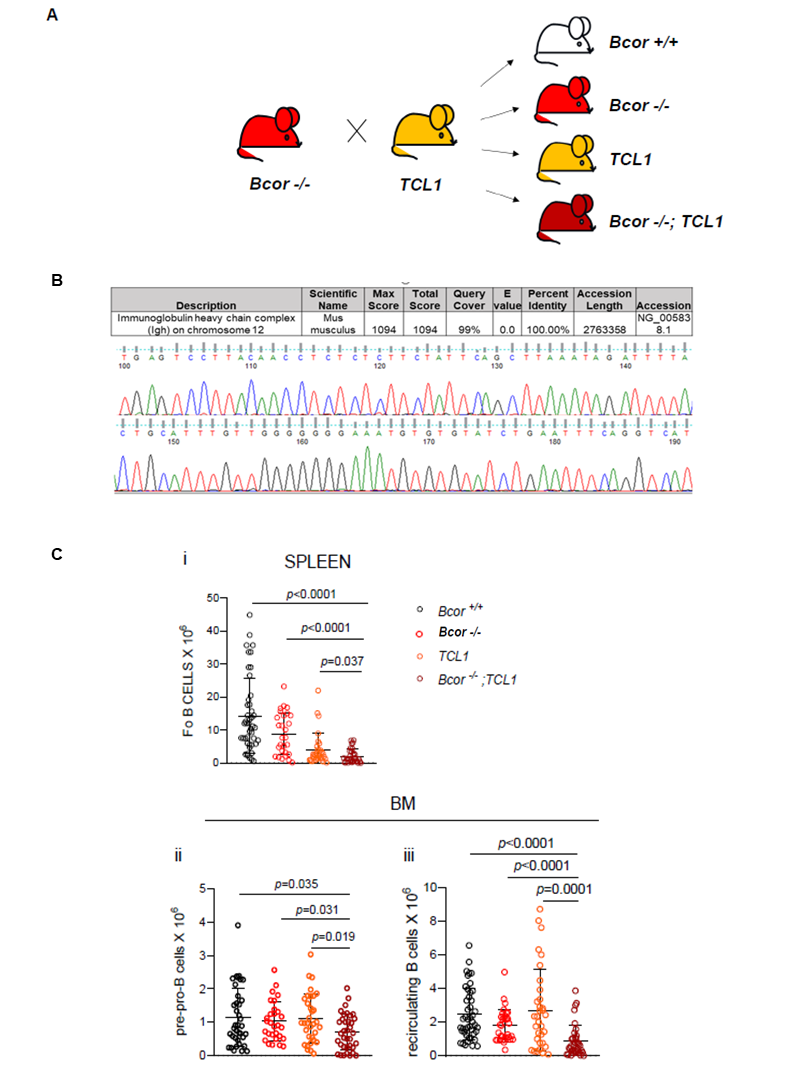
**

**Supplemental Figure 5**

(A) Mouse crossing strategy to generate murine genotypes used in this work. (B) Alignment results of the nucleotide sequence of *J558VH* gene (upper panel) amplification in splenic sorted CD19+CD5+ cells from one representative *Bcor^−/−^;TCL1* mouse, with the respective Sanger electropherogram (bottom panel), showing an unmutated clonal IGHV^1^ gene rearrangement (≥98% homology to germline). IgBlast Tool was used for the alignment of sequence against NCBI database. (C) Scatter dot plots graphs showing the differences in the number of: i) splenic Fo^2^ B cells from *Bcor^-/-^;TCL1* (2.08 x 106 ± 2.12 cells; N=31) compared to *Bcor^+/+^* (14.18 x 106 ± 11.38 cells; N=45), *Bcor^-/-^* (8.76 x 10^6^ ± 6.34 cells; N=28) and *TCL1* (4.00 x 10^6^ ± 4.89 cells; N=31) mice; ii) BM^3^ pre-pro-B cells from *Bcor^-/-^;TCL1* (0.70 x 10^6^ ± 0.53 cells; N=36) compared to Bcor^+/+^ (1.15 x 10^6^ ± 0.88 cells; N=37), *Bcor^-/-^* (1.03 x 10^6^ ± 0.59 cells; N=29) and *TCL1* (1.12 x 106 ± 0.73; N=33) mice; iii) BM^3^ recirculating mature B cells from *Bcor^-/-^;TCL1* (0.87 x 10^6^ ± 0.95 cells; N=36) compared to *Bcor^+/+^* (2.46 x 10^6^ ± 1.52 cells, N=45), *Bcor^-/-^* (1.80 x 10^6^ ± 0.95 cells; N=33) and *TCL1* (2.71 x 10^6^ ± 2.41 cells; N=33) mice.

^1^Immunoglobulin heavy chain variable region; ^2^Follicular; ^3^Bone Marrow.


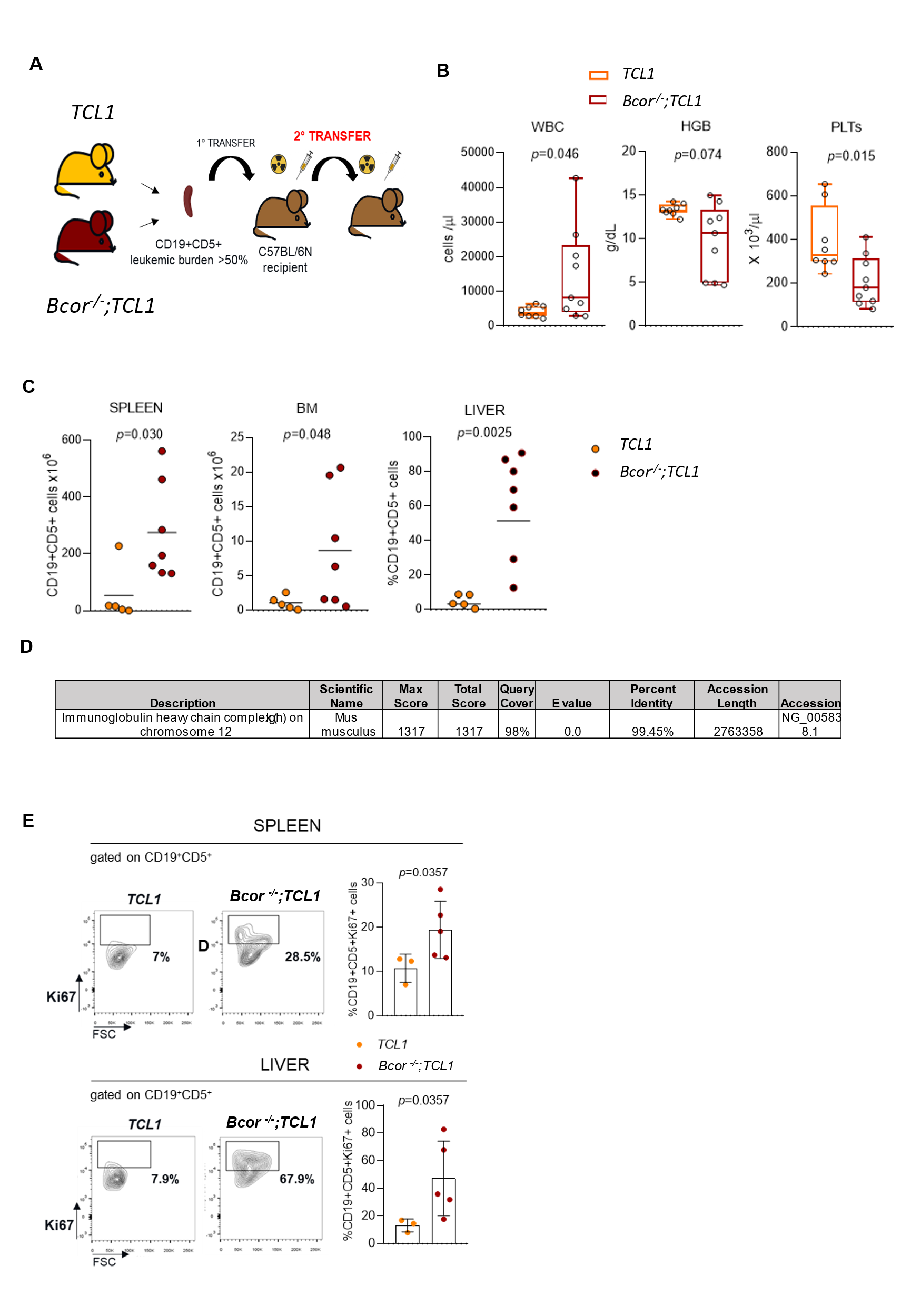


**Supplemental Figure 6**

(A) Schematic representation of the AT^1^ procedure: fully leukemic total spleen from one original leukemic mouse donor of *TCL1* or Bcor^-/-^*; TCL1* strain was I.V.^2^ injected into immunocompetent C57BL/6 recipients, after a sub-lethal total body irradiation. The transplanted mice used in the experiments and analysed derived from the second round of serial transplantation. (B) Box and whiskers graphs showing differences between PB^3^ from *TCL1* (N=8) and *Bcor^-/-^; TCL1* (N=9) transplanted mice in WBC^4^ count, HGB^5^ values and number of PLTs^6^. (C) FC^7^ analysis of CD19+CD5+ cells in transplanted *TCL1* (N=5) *vs* *Bcor^-/-^; TCL1* (N=7) mice in spleen, BM^8^ and liver. *P* values are indicated above each graph according to Mann-Whitney U test. (D) Alignment results’ of the nucleotide sequence of *J558VH* gene amplification in splenic sorted CD19+CD5+ cells from one representative Bcor^-/-^*; TCL1* transplanted mouse after Sanger sequencing analysis, showing an unmutated clonal IGHV^9^ gene rearrangement (≥98% homology to germline). IgBlast Tool was used for the alignment of sequence against NCBI database. (E) Representative result of FC^7^ dot plots (left) and scatter dot plot graph (right) showing Ki67 intra-cellular expression, gated on CD19+CD5+ cells, in spleen and liver of *Bcor^−/−^;TCL1* (N=5) compared to *TCL1* (N=3) transplanted mice.

^1^Adoptive Transfer; ^2^Intravenously; ^3^Peripheral Blood; ^4^White Blood Cells; ^5^Hemoglobin; ^6^Platelets; ^7^Flow Cytometry; ^8^Bone Marrow; ^9^Immunoglobulin heavy chain variable region

**
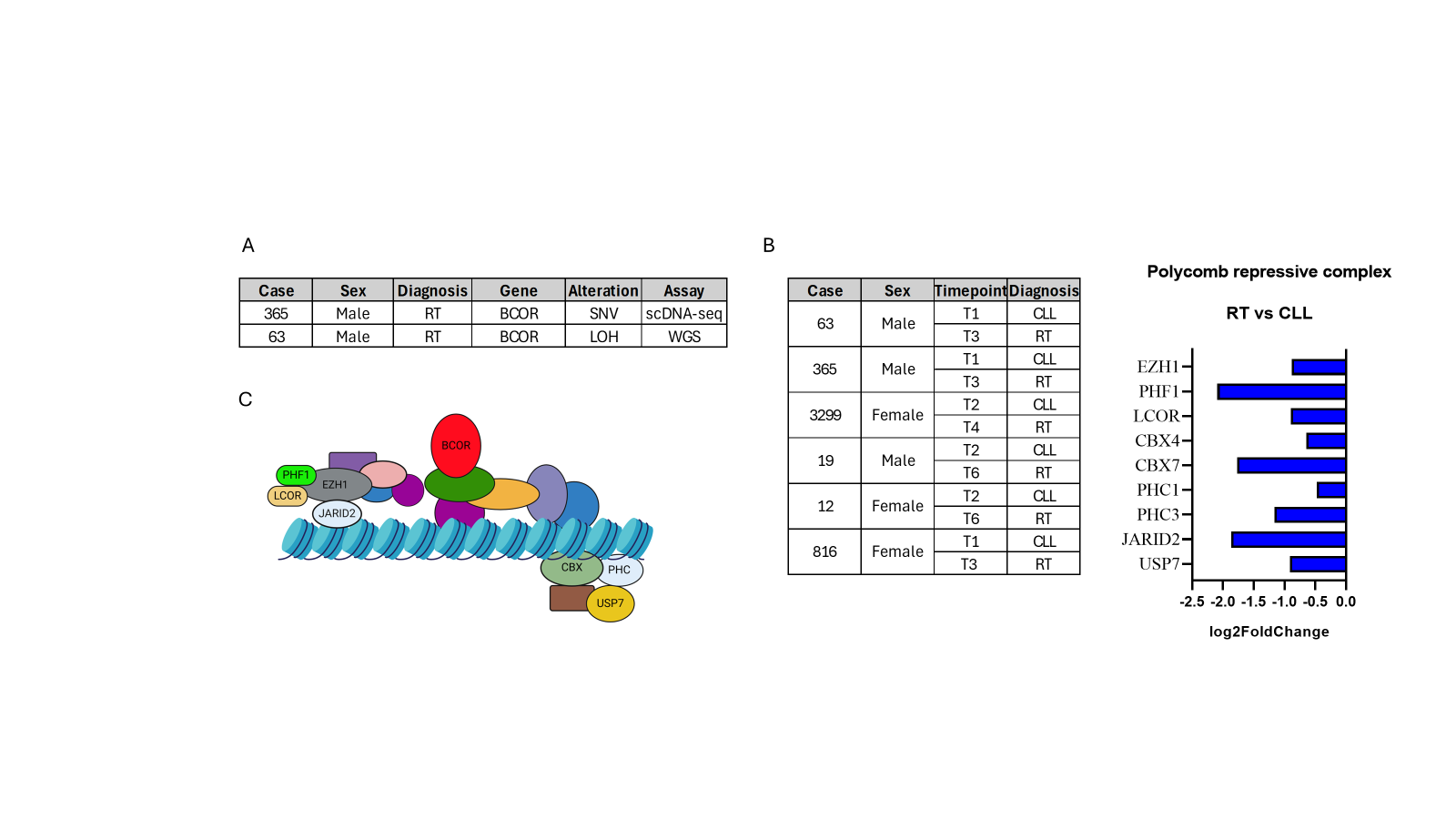
**

**Supplemental figure 7**

1. Identification of *BCOR* mutation and loss of heterozygosity LOH at the *BCOR* locus on chromosome X in RT^2^ patients (case 365 and 63) identified through single-cell DNA sequencing (scDNA-seq) and copy number alteration analysis (performed using WGS^3^) respectively, from the dataset no. [EGAS00001006327](https://ega-archive.org/studies/EGAS00001006327) (<http://www.ebi.ac.uk/ega/>)
2. Bar graph showing log2 Fold changes (*p*<0.05) of target genes of the polycomb repressive complex in RT^2^ patients (n=6) compared to the CLL^4^ stage (n=6), from the dataset no. [EGAS00001006327](https://ega-archive.org/studies/EGAS00001006327) (<http://www.ebi.ac.uk/ega/>)
3. Graphic representation of the polycomb repressive complex

^1^Loss Of Heterozygosity; ^2^Richter Transformation; ^3^Whole Genome Sequencing; ^4^Chronic Lymphocytic Leukemia
